# The shaping of genetic variation in edge-of-range populations under past and future climate change

**DOI:** 10.1111/ele.12158

**Published:** 2013-07-26

**Authors:** Orly Razgour, Javier Juste, Carlos Ibáñez, Andreas Kiefer, Hugo Rebelo, Sébastien J Puechmaille, Raphael Arlettaz, Terry Burke, Deborah A Dawson, Mark Beaumont, Gareth Jones, John Wiens

**Affiliations:** 1 School of Biological Sciences, University of BristolWoodland Rd., Bristol, BS8 1UG, UK; 2 NERC Biomolecular Analysis Facility, Animal and Plant Sciences, University of SheffieldWestern Bank, Sheffield, S10 2TN, UK; 3 Estación Biológica de Doñana (CSIC)Apdo 1056, 41080, Sevilla, Spain; 4 Department of Biogeography, Trier UniversityD-54286, Trier, Germany; 5 CIBIO, Centro de Investigação em Biodiversidade e Recursos Genéticos da Universidade do PortoCampus Agrário de Vairão, R. Padre Armando Quintas, 4485-661, Vairão, Portugal; 6 School of Biology and Environmental Sciences, University College DublinBelfield, Dublin, Ireland; 7 Institute of Ecology and Evolution, Division of Conservation Biology, University of Bern3012, Bern, Switzerland

**Keywords:** Approximate Bayesian computation, Chiroptera, ecological niche modelling, niche conservatism, phylogeography

## Abstract

With rates of climate change exceeding the rate at which many species are able to shift their range or adapt, it is important to understand how future changes are likely to affect biodiversity at all levels of organisation. Understanding past responses and extent of niche conservatism in climatic tolerance can help predict future consequences. We use an integrated approach to determine the genetic consequences of past and future climate changes on a bat species, *Plecotus austriacus*. Glacial refugia predicted by palaeo-modelling match those identified from analyses of extant genetic diversity and model-based inference of demographic history. Former refugial populations currently contain disproportionately high genetic diversity, but niche conservatism, shifts in suitable areas and barriers to migration mean that these hotspots of genetic diversity are under threat from future climate change. Evidence of population decline despite recent northward migration highlights the need to conserve leading-edge populations for spearheading future range shifts.

## Introduction

The effects of future climate change on biodiversity have been the focus of much research (Bellard *et al*. 2012), highlighting the potential extent of global species losses (Thomas *et al*. 2004) and documenting changes to the distribution of species (Parmesan & Yohe 2003). Recent estimates of the rate of global climate change suggest that many species may not be able to shift their range fast enough to track suitable conditions (Loarie *et al*. 2009), and therefore species survival will depend on phenotypic plasticity or adaptive capacity (Hoffmann & Sgrò 2011). However, despite the importance of genetic diversity for species persistence and adaptive capacity, genetic effects are often ignored in climate change studies (Pauls *et al*. 2013). Building on the integrated framework in Dawson *et al*. (2011), we predict the effects of future climate on patterns of genetic diversity across a species' range. We assess sensitivity and exposure to climate change and analyse how historical processes and barriers to movement shape the current distribution of genetic variation.

Quaternary climatic fluctuations, in the form of recurring glacial-interglacial cycles, contributed to the contemporary distribution and genetic composition of biodiversity (Hewitt 2000). In particular, stable populations that persisted from the Last Glacial Maximum (LGM ∼ 21,000BP) to the present harbour disproportionately large amounts of unique genetic diversity (Hampe & Petit 2005). Many European species survived periods of glaciation in southern refugia near the Mediterranean, from where they colonised their northern range during warmer interglacial periods (Hewitt 2000). Across the rest of the range, rapid climatic cooling and warming at the beginning and end of glaciation cycles were accompanied by a loss of genetic diversity and extinction of populations that were unable to track suitable conditions (Hofreiter & Stewart 2009). These losses provide warnings of the potential effects of future climate change, especially as projected rates of future changes dramatically exceed the past rates under which the climatic niches of vertebrates evolved (Quintero & Wiens 2013).

Bats are important indicators of responses to climatic changes due to their high diversity, wide distribution, high sensitivity to temperature change and keystone ecological roles (Jones *et al*. 2009). Extant populations of European bats show a strong genetic signature of range expansion from Mediterranean glacial refugia (e.g. Rebelo *et al*. 2012). This pattern is common for European temperate biodiversity, ranging from plants to mammals, and has resulted in high concentrations of genetic diversity in Iberia, Italy and the Balkans (Hewitt 2000). Hence, the analysis we present here is likely to be relevant for many temperate organisms that were forced to contract away from the poles into glacial refugia during the LGM.

The longevity and slow reproductive rates of bats suggest that they may not be able to evolve fast enough to respond to future changes because of the slow spread of favourable traits through the population (Hoffmann & Sgrò 2011).Yet, there is a paucity of studies investigating potential effects of future climate change on bats, and in particular potential genetic consequences (Sherwin *et al*. 2013). The few published studies to date show that temperate species are likely to experience distributional shifts and range contractions, with some species losing their entire suitable niche space (Rebelo *et al*. 2010).

We studied the effects of historic and future climatic changes on the spatial distribution of genetic variation in the grey long-eared bat, *Plecotus austriacus*, a medium-sized insectivorous bat distributed from the northern Mediterranean to central Europe (Spitzenberger *et al*. 2006). *P. austriacus* is considered to be at relatively high risk from the effects of climate change due to its limited vagility and restricted distribution compared with other bat species (Sherwin *et al*. 2013). Although the ability to fly means that bats may be able to shift their range in response to climatic changes more readily than other small mammals, dispersal is more limited in bats with wing morphologies that place high energetic costs on long distance flight (Norberg & Rayner 1987). As such these bats can offer meaningful estimates of the effects of climate change on biodiversity in general, including non-volant organisms.

Evolutionary processes shaping the genetic properties of individuals, populations and species are often stochastic and complex. Therefore, understanding these processes necessitates the use of probabilistic models with several interdependent parameters (Beaumont & Rannala 2004). Approximate Bayesian Computation (ABC) is widely used in population genetics and phylogeography to reconstruct demographic history under complex evolutionary scenarios in a Bayesian setting, without the need to compute the likelihood function (Bertorelle *et al*. 2010). The inference is based on large sets of stochastic simulations, parameters sampled from a probability distribution and summary statistics that capture information in the data (Csilléry *et al*. 2010).

Ecological Niche Models (ENMs), on the other hand, are used to predict the current and future distribution of species based on their environmental requirements (reviewed in Elith & Leathwick 2009). When hindcasting the past (palaeo-ENMs), these models can help identify the location of glacial refugia and provide prior information for parameterising evolutionary and demographic models (Knowles & Alvarado–Serrano 2010). Despite their limitations due to lack of incorporation of population dynamics, dispersal rates or biotic interactions, ENMs greatly contributed to our understanding of factors affecting the current distribution of species and the potential effects of future climate change on biodiversity (Botkin *et al*. 2007).

Projecting the effects of future climate change on biodiversity is complicated by the fact that a species' current climatic niche does not necessarily reflect future climatic tolerance (Guisan & Thuiller 2005). Therefore, before predicting the effects of future climates on the distribution of species, it is recommended to assess whether their climatic niche remained similar over time. Such niche conservatism will result in geographical range shifts to track the distribution of a specific set of climatic conditions (Wiens *et al*. 2010). Niche conservatism can be tested by validating with genetic data the inference of palaeo-ENMs on the location of glacial refugia (Cordellier & Pfenninger 2009). This phylogeographical approach is an extension of the paleontological approach, which looks for range shifts in response to changing climates (habitat tracking) in the fossil record (Wiens *et al*. 2010), and is particularly relevant for groups of species, like bats, that are underrepresented in the fossil record (Teeling *et al*. 2005).

We combined phylogenetic analysis and ABC inference of evolutionary history with ENMs across temporal scales to test the hypothesis that the niche of *P. austriacus* has been conserved in term of climatic tolerance and therefore can be projected into the future to predict the genetic consequences of climate change. We also assess the potential for future range shifts given the presence of geographical barriers to movement, current patterns of gene flow and recent changes in population size. Our overall aim is to show how current genetic diversity has been shaped by climate change in the past, and to predict how future patterns of genetic diversity may be affected by contemporary climate change. We are especially interested in determining whether current hotspots of genetic diversity resulting from past climate changes are those most at risk from future climate change.

## Material and Methods

### Sample collection and laboratory procedures

The 259 *P. austriacus* genetic samples included in this study were collected or obtained from 82 geographical locations spanning the entire known species' range, representing six main geographical populations separated by either mountain ranges or extensive water [England: *n* = 54, Channel Isles: *n* = 24, Mainland Western Europe (France, Belgium, Germany, Switzerland): *n* = 60, Iberia: *n* = 91, Italy (including Corsica and Sardinia): *n* = 16, Balkans: *n* = 14; Table S1, Fig. 3a].

Genomic DNA was extracted from all samples and amplified for one mitochondrial (mtDNA) gene (747bp of Cytochrome *b* [*Cyt b*]) and genotyped at 23 autosomal microsatellite loci specifically designed for this study (Table S2). Microsatellite library design, mtDNA primers, PCR reaction conditions, PCR cycle programs and microsatellite loci selection procedures are outlined in Appendix S1.

### Genetic analysis

We constructed Bayesian phylogenetic trees for the mtDNA region, using sequences of two congenerics (*Plecotus auritus*, downloaded from GenBank AY665169, and *Plecotus macrobullaris,* provided by Antton Alberdi) and one species of the same family (*Myotis bechsteinii*, AF376843) as outgroups to root the tree (Appendix S1). Parsimony haplotype networks for the *Cyt b* sequences were constructed using the program NETWORK (v4.610, http://www.fluxus-engineering.com). See Appendix S1 for analysis of mtDNA and microsatellite genetic diversity.

Individual-based Bayesian assignment tests implemented in STRUCTURE v2.3.3 (Pritchard *et al*. 2000) were used to infer population structure in the microsatellite data set, varying the number of genetic clusters (K) between 1 and 13, and performing ten independent runs for each K. We used the general admixture model with correlated allele frequencies and 10^6^ Markov Chain Monte Carlo (MCMC) generations following a burn-in phase of 5 × 10^5^ generations (Appendix S1).

We used the Bayesian approach implemented in BayesAss v3 (BA3) (Wilson & Rannala 2003) to estimate contemporary gene flow (within the last few generations) and the direction of migration between the main geographical areas. We adjusted the MCMC mixing parameter values of migration rates, allele frequencies and inbreeding coefficients to obtain the recommended acceptance rates for proposed changes. We performed five replicate runs with 10^7^ MCMC iterations and a burn-in phase of 10^6^ iterations, initializing each run with a different random number generator seed to verify convergence based on concordance between runs on the posterior mean parameter estimates.

### Ecological niche modelling procedure

ENMs were generated with the program Maxent (Phillips *et al*. 2006) to determine the potential distribution of *P. austriacus* across its range under present, past [LGM ∼21,000BP, and the Last Interglacial period (LIG) ∼130,000BP] and future (2080) climatic conditions. Models were run at a resolution of approximately 5 km (2.5 arc min). The study extent was set as the whole of Europe including Mediterranean islands, because the Mediterranean Sea represents the southern limit of the species' distribution (Spitzenberger *et al*. 2006).

Climatic and topographic environmental layers were downloaded from World Clim (http://www.worldclim.org). After removing highly correlated variables (correlation coefficients ≥0.8) and variables that did not contribute to the ENMs, the following environmental variables were included in the models: mean temperature of the coldest quarter (BIO11), temperature annual range (BIO7), annual mean temperature (BIO1), annual precipitation (BIO12), slope, altitude, precipitation during the warmest quarter (BIO18) and mean temperature of the warmest quarter (BIO10).

We ran 50 replicates of each model, each time randomly selecting 80% of locations to train the models and 20% to test them. The 50 replicates were averaged into a single model. Model performance was evaluated based on the Area under the Curve (AUC) of the Receiver Operator Characteristics (Appendix S1).

Models were projected into the past using the CCSM and MIRCO General Circulation Models (GCMs) for the LGM and one LIG model (WorldClim http://www.worldclim.org). Areas that were predicted to remain suitable across LIG, LGM and current conditions were assigned as potential glacial refugia.

Future projections for 2080 were performed with three GCMs: HadCM3, IPSL-CM4 and CCSM (GCM data portal http://www.ccafs-climate.org/), using the A2 scenario (Appendix S1).

The results of future ENMs were used to estimate extent of genetic diversity losses based on the number of unique haplotypes and private alleles that will be found by 2080 in areas with unsuitable conditions. We assessed the accuracy of our estimate of haplotype diversity (degree of sampling completeness) using Species Accumulation Curves (Appendix S1), based on the approach outlined in Pfenninger *et al*. (2012).

### ABC inference of demographic history

We reconstructed the evolutionary and demographic history of *P. austriacus* using the ABC approach implemented in DIYABC v1.0.4 (Cornuet *et al*. 2008, 2010). We carried out two sets of analyses aimed to infer the source population and patterns of post-LGM range colonisation from putative refugia locations identified in palaeo-ENMs. Scenarios compared in the first analysis included between one and three source populations (Iberia, Italy and Balkans) and range colonisation from a single refugial population or admixture between populations (Fig. S6a). A preliminary analysis identified that the northern edge-of-the range was colonised in a stepping-stone manner instead of long-range colonisation directly from glacial refugia (Appendix S1, Fig. S7). Building on the results of the first analyses, the second analysis included range colonisation from an unsampled refugial population from southern France either directly or through admixture with other populations, and the colonisation of the northern edge-of-the range (Fig. S6b).

In addition, we carried out a demographic history analysis of the northern edge English population to determine changes in population size since colonisation. We compared a null model of no change in population size to a model of a short bottleneck during colonisation followed by population expansion, and a model of recent change (increase or decrease) in population size (Fig. S8).

In all ABC analyses, we generated 10^6^ simulations for each scenario tested using the combined microsatellite and mtDNA data sets (Appendix S1 for model parameters). The posterior probability of scenarios was estimated using a weighted polychotomous logistic regression on the 1% of simulated data sets that were closest to the observed data set. Taking into account the criticism of ABC model choice outlined in Robert *et al*. (2011), we empirically evaluated the power of the model to discriminate among scenarios using a Monte Carlo estimation of false allocation rates (type1 and 2 errors) resulting from ABC posterior probabilities-based model selection (Cornuet *et al*. 2010).

### Testing for niche conservatism

We tested for niche conservatism by comparing the location of Pleistocene glacial refugia identified through palaeo-ENMs, phylogenetic analysis and ABC inference of demographic history. A match between predicted range of the species during the LGM based on its current climatic requirements and phylogeographical inference of LGM refugia can indicate that the climatic niche of the species was conserved over time (Cordellier & Pfenninger 2009). The ABC framework allowed us to statistically test whether ENM model predictions correspond to genetically determined model of LGM refugia and post-LGM range colonisation. We assume that the climatic tolerance of organisms to cold conditions represents their general tolerance to climatic changes and their tendency to track the distribution of a certain set of climatic conditions.

We conducted a MESS analysis in Maxent (Elith *et al*. 2010) to determine whether climatic conditions in genetically determined LGM refugia are similar to conditions present in the current range of the species and to identify the most dissimilar climatic variables between the two time periods.

## Results

### Current patterns of genetic variation

**Figure 1:**
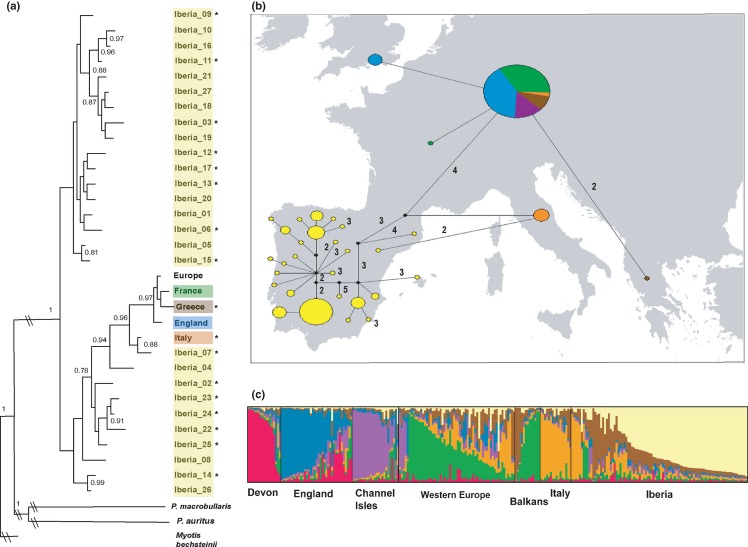
Population structure of *Plecotus austriacus* across its range. (a) Bayesian phylogenetic tree of *Cyt b* haplotypes showing posterior probability values > 0.75. Asterisks represent unique haplotypes that may be lost due to future climate change. Haplotypes (Table S5) are colour-coded based on geographical areas. (b) Median-joining network of *Cyt b* haplotypes, mapped based on the approximate location of samples and colour-coded based on their geographical areas. Circle size corresponds to number of samples, and numbers represent connections separated by more than one mutation. (c) Results of the STRUCTURE analysis separating the microsatellite data set into seven clusters, plotting individual samples based on their geographical location and cluster membership.

The phylogenetic analysis of the mtDNA gene (*Cyt b*) in *P. austriacus* identified 32 haplotypes across the species' range, 27 of which were confined to Iberian populations, which are located at the rear-edge-of-the range (Fig. 1a). The Iberian population also contained the highest microsatellite diversity and 76% of the private alleles identified in the species (Table 1). *P. austriacus* haplotypes were divided into two main clades (100% posterior probability support), referred to henceforth as the south-eastern and north-western clades. All samples from outside Iberia were nested within the south-eastern clade forming a subgroup with two eastern Iberian haplotypes located just south of the Pyrenees (Fig. 1a). The majority of samples from outside Iberia shared the common pan-European haplotype or have recently emerged from that haplotype following a star-like pattern (Fig. 1a), indicating rapid population expansion during colonisation. At the northern edge, the English population contained one unique mtDNA haplotype (Fig. 1b) and two private alleles, but levels of genetic diversity tended to be lower than in rear-edge populations (Table 1).

**Table 1:** Genetic diversity of *Plecotus austriacus* geographical populations based on 23 microsatellite loci (first three columns) and the *Cyt b* mtDNA gene (last two columns), with sample sizes presented in brackets. Mean allelic richness and gene diversity (± SD) were adjusted based on sample size

	Mean allelic richness	Mean gene diversity	No. of private alleles	mtDNA haplotype diversity	mtDNA nucleotide diversity
England (54)	4.89 ± 1.9	0.68 ± 0.2	2	0.14	0.0002
Channel Isles (24)	5.06 ± 1.9	0.68 ± 0.2	0	0	0
Western Europe (60)	5.59 ± 2.1	0.71 ± 0.2	9	0.11	0.0001
Italy (16)	5.47 ± 2.1	0.70 ± 0.2	3	0.49	0.0033
Iberia (91)	6.23 ± 2.3	0.74 ± 0.2	83	0.82	0.0066
Balkans (14)	5.73 ± 2.1	0.67 ± 0.2	12	0.17	0.0005

Genetic differentiation at the mtDNA level was significant among all populations, except those in Western Europe, England and the Channel Isles. Particularly, high levels of differentiation were found between Iberia and Italy and all other populations (θ_ST_ = 0.61–0.83; Table S3). At the microsatellite level, individual-based assignment tests detected genetic structure across the range of *P. austriacus*, dividing the samples into seven clusters (Fig. S1), roughly corresponding to the six main geographical areas separated by mountains ranges or large expanses of water. Mainland Western European samples, though belonging primarily to the Balkan cluster, showed high levels of admixture (Fig. 1c), corresponding to the high estimated gene flow into that population (Table S4).

Bayesian estimations of contemporary gene flow rates among the six geographical populations (expressed as proportions of the population migrating into other regions in the last few generations) indicate a predominately north-western migration direction across the range of *P. austriacus*. High rates of gene flow were estimated from Italy (0.24) and the Balkans (0.23) into Western Europe and from Western Europe into England (0.17), while rates of gene flow in the opposite (southern) direction were very low (< 0.01). We found limited contemporary gene flow across the Pyrenees both in and out of Iberia, with the majority of Iberian bats remaining in Iberia in the last few generations (94%, Fig. 3b, Table S4).

### Ecological niche modelling across temporal scales

**Figure 2:**
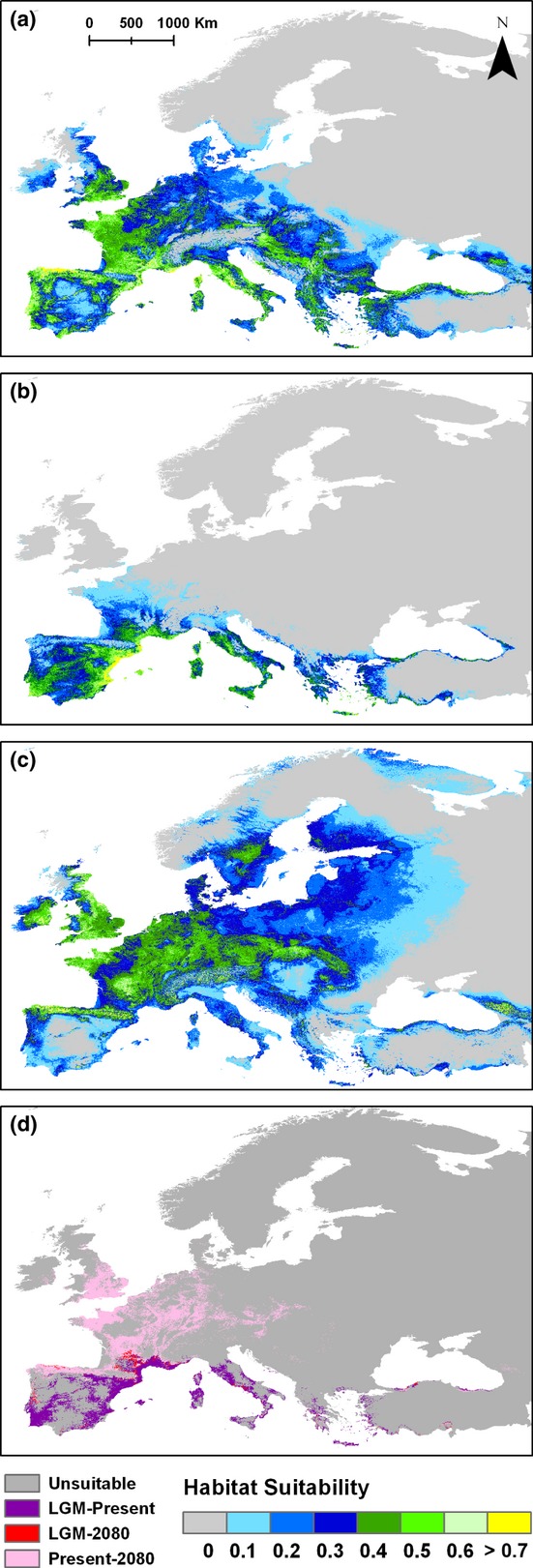
Effects of climate changes on range suitability across temporal scales. ENMs showing the predicted (a) current, (b) past Last Glacial Maximum (LGM ∼ 21 000BP) and (c) future (2080, A2 scenario) distribution of suitable conditions for *Plecotus austriacus*. Habitat suitability ranges between zero (unsuitable, in grey) and > 0.7 (highly suitable, in yellow), with green and yellow representing suitable areas. (d) Reclassified map of stable areas between the LGM and present (in purple), currently suitable areas that will remain suitable until 2080 (pink), and areas that will remain stable between the LGM and 2080 (red).

All models had high predictive ability and did not overfit presence data (AUC_training_ = 0.943 ± 0.01, AUC_test_ = 0.9 ± 0.02). The primary environmental variables affecting habitat suitability for *P. austriacus* across its range were winter temperature, temperature range, annual temperature and annual rainfall. Palaeo-ENMs predicted that suitable climatic conditions for *P. austriacus* during the LGM occurred in Iberia, Italy and the Mediterranean coast of France and the Balkans, but highly suitable conditions (habitat suitability > 0.7) only existed along the east coast of Iberia (Fig. 2b). Suitable conditions persisted in Iberia, Italy and southern France from the LGM till the present and between the LIG and the LGM, suggesting that these areas had potential to act as refugia across Pleistocene glaciation cycles (Fig. 2d_,_ Fig. S2).

A MESS analysis showed that climatic conditions in the whole of Iberia during the LGM had parallels to conditions found within the current range of *P. austriacus*. In contrast, northern, western and central Europe experienced during the LGM summer temperatures, annual temperatures and temperature ranges that are outside the current climatic niche of the species (Fig. S3).

Future ENMs predict that climate change will result in a north-western shift in the distribution of *P. austriacus*, with suitable conditions restricted to northern parts of Iberia, areas north of the Alps and to the north-west of the Balkans (Fig. 2c). The majority of stable areas from the LGM, in particular in Iberia and Italy, are likely to become unsuitable by 2080 (Fig. 2d). Based on the results of a MESS analysis, by 2080, most of Iberia as well as the Mediterranean coast of Italy and the Balkans will experience novel climatic conditions, currently not present in the species range, in particular in terms of annual and winter temperatures (Fig. S4).

This will result in more than half of the species' genetic diversity (53% of haplotypes and 58% of private alleles) being located in unsuitable areas or small isolated patches of suitable habitats. Losses of genetic diversity are predicted to be particularly high in Iberia (Fig. 1a). Accumulation Curves confirmed that our sampling at the haplotype level was complete because the resampling curve reached saturation (Fig. S5), showing that the sampled number of haplotypes is an accurate reflection of the total number of haplotypes in the sampling area.

### Model-based inference of demographic history

**Figure 3:**
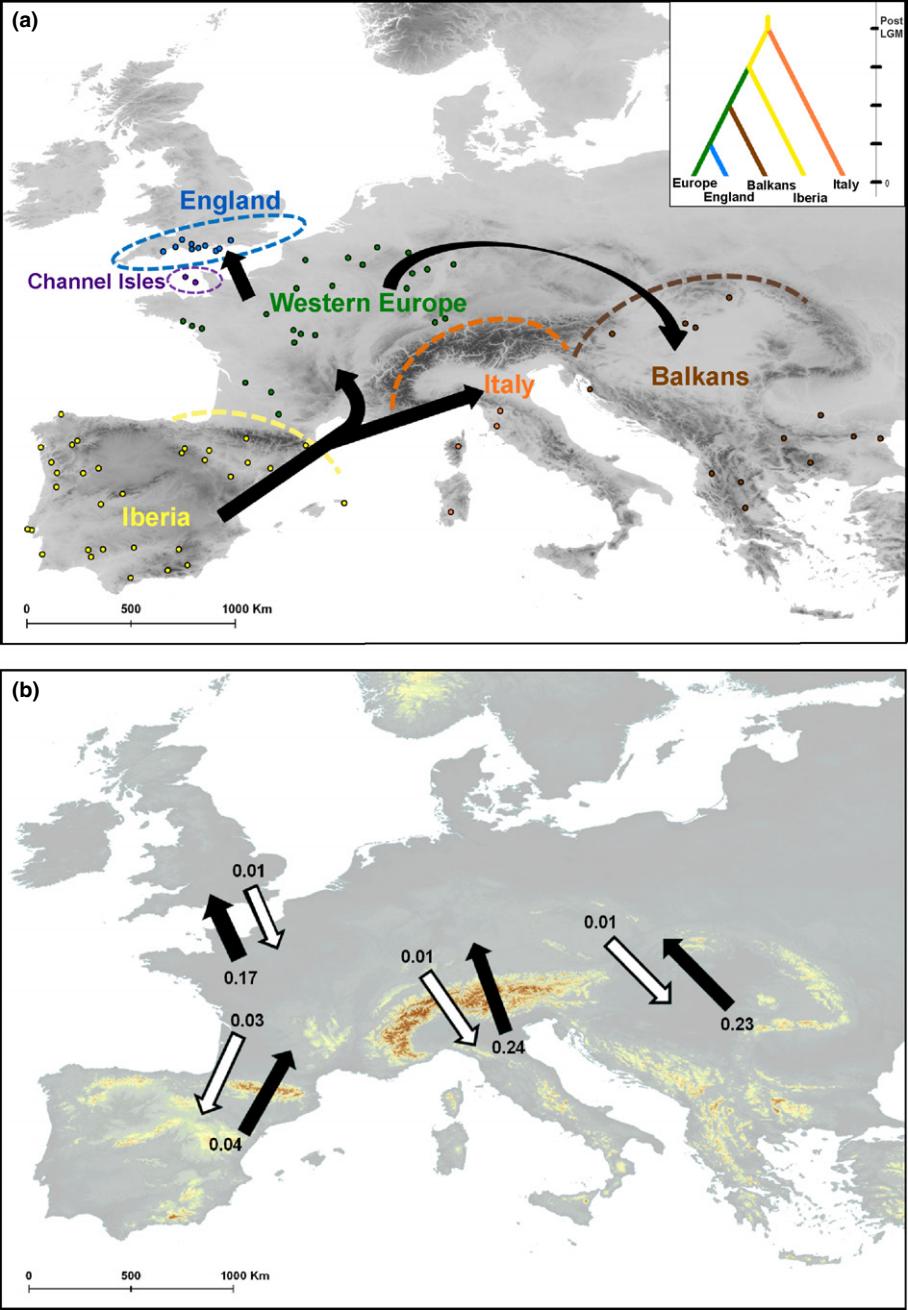
Patterns of movement across the range of *Plecotus austriacus*. (a) The geographical location of *P. austriacus* genetic samples included in the study plotted over an elevation map, with the location of the six populations marked and colour coded. Arrows indicate patterns of post-Last Glacial Maximum range colonisation from the Iberia refugium based on ABC model inference. The selected demographic history scenario is presented in the insert. (B) Estimates of contemporary gene flow plotted over an elevation map, showing the proportion of the population that migrated in the direction of the arrow in the last few generations (black arrows – northern migration, while arrows – southern migration).

Model-based inference pointed to Iberia being the main Pleistocene refugium for *P. austriacus* and the source population from which all other European populations emerged (Fig. 3a). The Iberian source population scenario was supported with maximum probability relative to around zero support for scenarios including Italian, Balkan and unsampled refugia in southern France (Fig. S6). Confidence in scenario choice was high, with low error rates (type 1 = 0.01–0.02; type 2 = 0.01–0.05). Post-LGM range colonisation followed a stepping-stone model of north of range colonisation (Fig. S6a, S7), whereby Western Europe and Italy were colonised directly from Iberia, while the Balkans and at a later stage England were colonised from Western Europe (Fig. 3a).

Within the northern edge-of-the range, ABC inference indicated a recent trend of decline in the English population with the current effective population size estimated at more than 30-fold smaller than the historic size. Support for the recent decline scenario was high (around 90%) relative to zero support for the bottleneck scenario and around 10% support for the no change in population size model (Fig. S8). Error rates were estimated at 0.14 and 0.04 for type 1 and 2 errors respectively.

## Discussion

This study highlights the importance of combining a historic perspective with future predictions to understand the genetic consequences of climate change. We show that historic climate changes and geographical barriers played an important role in shaping patterns of genetic variation in *P. austriacus*, and that future climate change is likely to reshape these patterns resulting in extensive losses of genetic diversity and pose the greatest threat to former refugial populations with the highest genetic diversity. This pattern is likely to be common to many temperate European species that survived Pleistocene glaciation events in Mediterranean refugia.

### Reconstructing the historic distribution of genetic variation

ABC demographic history inference indicates that Iberia was the main Pleistocene glacial refugium of *P. austriacus* and that all other populations emerged from the Iberian population, an inference supported by palaeo-modelling and the phylogenetic analysis. Evidence from the fossil record also supports the Iberian origin of *P. austriacus*, and suggests that it did not appear in central Europe before the mid-Holocene (Juste *et al*. 2004). In agreement with the ABC inference, all non-Iberian samples are located within the south-eastern Iberian clade and the haplotype network shows a single star-shaped, rapid population expansion event across Europe. Palaeo-ENMs for the LIG confirm that suitable conditions for *P. austriacus* existed in Iberia for at least 130 000 years, thus suggesting that Iberia represents a ‘stable rear-edge’ population (Hampe & Petit 2005).

Iberia harbours substantially higher levels of mitochondrial and nuclear DNA diversity than the rest of the range and the greatest number of unique haplotypes and private alleles, a further testimony to the long-term stability of Iberian populations (Hewitt 2000). The persistence of stable populations in parts of Iberia throughout a full series of glacial-interglacial cycles, combined with disproportionately higher levels of genetic diversity, indicate that this long-term refugium is of high evolutionary importance (Stewart *et al*. 2010).

Despite being recognised as an important glacial refugium for most European bat species studied so far (e.g. Rebelo *et al*. 2012), the Balkans were not identified as the source population of *P. austriacus*. This may be due to the presence of a cryptic sister species, *Plecotus kolombatovici*, in the area (Juste *et al*. 2004). Similarly, Italy was not identified as an LGM refugium for *P. austriacus* despite the predicted suitable conditions in Italy during the LIG and LGM and despite the presence of a unique Italian mtDNA haplotype and microsatellite cluster. Absence of sufficient samples from the Mediterranean coast of France meant that inference regarding the role of this potential refugium could only be made by including a ghost, unsampled population in the analysis.

Concordance between the location of glacial refugia identified in this study through genetic analysis and ecological niche modelling indicates that the climatic niche of *P. austriacus* is likely to be conserved and therefore can be projected to estimate habitat suitability under future climate change (Cordellier & Pfenninger 2009). Moreover, the MESS analysis confirmed that climatic conditions experienced by *P. austriacus* in Iberia during the LGM are similar to current conditions found across its range. Although niche conservatism in terms of tolerance to cold is not necessarily an indication of future tolerance to warming conditions, rates of future changes may be too fast for the climatic niche to evolve (Quintero & Wiens 2013).

### Forecasting effects of future climate change

A species' ability to respond to future climate change depends on intrinsic factors, including physiological sensitivity to changes, genetic adaptive capacity and dispersal ability, the latter being affected by extrinsic factors such as geographical barriers (Dawson *et al*. 2011). Despite their high potential for dispersal by flight, bat species show different patterns of population structure due to differences in movement abilities, migration and mating behaviour (Burland & Worthington Wilmer 2001). In particular, species with wing morphologies that limit long-distance flight, like *P. austriacus* (Norberg & Rayner 1987), are potentially less able to respond to climate-induced range shifts and tend to be generally more vulnerable to extinction (Safi & Kerth 2004).

In line with previous broad-scale predictions for other temperate European bat species (Rebelo *et al*. 2010), our models predict northern range expansion and southern range contraction by the end of the century. Increase in temperatures and aridity around the Mediterranean is predicted to result in most stable LGM refugial areas becoming climatically unsuitable for *P. austriacus*. These predictions are of great concern given the genetic impoverishment evident in northern parts of the range of *P. austriacus,* and the genetic evidence from an ancient DNA study questioning the ability of species to track decreases in availability of suitable habitats under climate change (Dalén *et al*. 2007). Widespread range retraction and population extinctions relating to recent climatic changes are already evident in several butterfly and frog species (Thomas *et al*. 2006). While losses of genetic diversity and the disappearance of evolutionary lineages are predicted for other European taxa (Bálint *et al*. 2011; Provan & Maggs 2012); here, we show that losses are likely to be most extensive where genetic diversity is highest.

It has been debated whether ENM predictions relate to the realised rather than fundamental niche of species, and as such reflect the effects of barriers to colonisation and interspecific interactions rather than the environmental tolerance of species (Elith & Leathwick 2009). However, we believe that in our study the modelled niche of *P. austriacus* indeed represents its climatic tolerance. *P. austriacus* is absent from North Africa and the Middle East (Spitzenberger *et al*. 2006), where climatic conditions are more arid, despite its ability to colonise islands separated by larger expanses of water than the Gibraltar Straits (García Mudarra *et al*. 2009) and the fact it is found in sympatry with other *Plecotus* species across Europe. Moreover, differences between the fundamental and realised niche may be generally small in bats because flight allows bats to disperse widely and colonise the entire potential geographical niche space (Rebelo & Jones 2010).

The predominantly north-western direction of current gene flow in *P. austriacus*, from Italy and the Balkans into Western Europe and from Western Europe into England, may already reflect ongoing northward shifts in distribution in response to climate change. However, despite the relatively high rates of estimated gene flow into the English population, we found evidence of a recent decline in population size. This trend of decline suggests that although favourable climatic conditions increased in the past few decades at the northern edge, other factors like habitat loss and modification may limit future range expansions, as has already been shown to be the case for northern-edge butterfly populations in the United Kingdom (Warren *et al*. 2001).

Availability of suitable climatic conditions at the northern edge does not imply that the ecological conditions necessary for the colonisation and formation of viable populations will be present (Pearson & Dawson 2003). Therefore, ENM predictions may be an over-estimation of future range suitability. Beyond dispersal limitations, population expansion in intensively farmed areas, like England, may be limited by the scarcity of suitable foraging habitats such as unimproved grasslands (Razgour *et al*. 2011).

Limited contemporary gene flow across the Pyrenees indicates that this mountain barrier may impede migration out of Iberia. The Pyrenees are likely to remain a barrier for the dispersal of *P. austriacus* even if climate change will result in warmer conditions because of their physical structure and the role of altitude in limiting the distribution of this species. *P. austriacus* is commonly found at low elevations (Spitzenberger *et al*. 2006), and its limited long-distance flight ability suggests that crossing such physical barriers may be a rare event. Indeed, the current low genetic diversity outside Iberia and the emergence of all European haplotypes from a single north-eastern Iberian haplotype suggests that during the Holocene, when this species expanded its range out of Iberia, only a limited number of individuals from a population adjacent to the Pyrenees crossed this geographical barrier.

Due to the Pyrenees barrier, much of Iberian genetic diversity may become ‘locked’ inside the peninsula, and population persistence will depend on the phenotypic plasticity or genetic adaptability of Iberian populations. However, evidence of niche conservatism, combined with a slow reproductive rate and a long lifespan, suggest that *P. austriacus* may have limited ability to evolve fast enough to respond to rapid climatic changes (Hoffmann & Sgrò 2011), especially as adaptations may require unprecedented rates of climatic niche evolution (Quintero & Wiens 2013). Hence, population extinctions and loss of around half of the species' genetic diversity may be a likely outcome, especially given the increased fragmentation and isolation of remaining suitable areas, factors already implicated in the recent climate-induced genetic erosion of an alpine mammal (Rubidge *et al*. 2012). These losses are particularly concerning because of the importance of stable refugial populations as long-term reservoirs of genetic diversity for species survival and evolution (Hampe & Petit 2005). Given the relatively high dispersal potential of bats, genetic consequences of climate change for non-volant species may be even more severe.

## Conclusions

This study provides a new slant on the importance of conserving edge-of-range populations. We show that former refugial populations that contain the highest genetic diversity due to past climate change are highly endangered due to future climate change. These predictions are relevant to much of European biodiversity that displays similar phylogeographical patterns, and therefore may experience a similar fate. Conservation efforts should also recognise the importance of leading-edge populations, regardless of their current size, due to their role in range shifts and the future spread of genetic diversity. Evolution in marginal atypical environments may allow edge populations to better respond to environmental changes and expand into new habitats, and therefore lead range shifts in response to climate change (Hunter & Hutchinson 1994). However, range shifts and successful population establishment depend on the availability of suitable habitats in the industrialised, urbanised and intensive agricultural landscape of north-western Europe. Therefore, the conservation of edge populations is integral for ensuring the long-term maintenance of temperate genetic diversity.
