# Supplementary material for: The shaping of genetic variation in edge-of-range populations under past and future climate change
Source: Ecol Lett. 2013 Jul 26;16(10):1258–66. doi: 10.1111/ele.12158 (PMC4015367; doi:10.1111/ele.12158)
Supplement: Supplementary file 1 [file ele0016-1258-sd1.doc]

# Appendix S1

## Mitochondrial DNA sequencing

We sequenced fragments of mitochondrial DNA (mtDNA) located within the *Cytochrome* *b* (*Cyt* *b*) gene, using a new primer set designed with the program Primer3 (Rozen & Skaletsky 2000) based on a consensus sequence of five *Plecotus austriacus* sequences from Iberia (EMBL accession numbers: AF513774, AF513776, AF513786, AF513792, AF513793) combined with a *P. austriacus* sequence from England (S. L. Harris *pers. comm*.) to create a 942 base pair (bp) sequence. The designed primer pair, RazgourCytbF (5’-TTGATCTCCCTACCCCTTCA-3’) and RazgourCytbR (5’-CTCCGCCCAGTTTATTAGGA-3’), was used to amplify 747 bp of the *Cyt b* gene. PCRs were performed in 20μL reactions containing 2μL of DNA extraction (<10ng), 10μl of Qiagen HotStar plus PCR master mix, 1μl of each primer (concentration 0.1µM) and 2μl of loading buffer (Coraload, Qiagen).

PCR cycle program: initial denaturation at 95°C for 5 min, followed by 35 cycles of 94°C for 30 s, 57°C for 30 s and 72°C for 60 s, followed by a final extension at 72°C for 10 min and 4°C forever. 5 µl of each PCR product was visualized on a 1.5% agarose gel stained with Web Green DNA stain (Web Scientific). When amplification was observed on the gel, PCR-products were sent to LGC Genomics (Germany) for purification and sequencing.

Sequences were edited using Bioedit v7.1.3 (Ibis Biosciences), aligned with Muscle (Edgar 2004), and collapsed into unique haplotypes with Dambe v5.2.31 (Xia & Xie 2001). Because this species is part of cryptic species complex, a BLAST search of the NCBI GenBank nucleotide dataset was performed on all haplotypes to confirm species identification.

## Microsatellite genotyping

We tested a library of 36 nuclear microsatellite loci (EMBL accession numbers HE983997–HE984032) constructed for this study using 454 high-throughput sequencing (Roche GS-FLX), following the methods described in Alfadala *et al*. (2012). We also included three previously published *Plecotus auritus* microsatellite loci (Burland *et al*. 1998) that are known to be polymorphic in *P. austriacus* (*Paur01*, *Paur05* and *Paur06*).

The forward primer of each locus pair was labeled fluorescently with HEX or 6-FAM (Applied Biosystems). Microsatellites that were polymorphic, amplified well and easily scorable in initial tests on 26 samples from across the range were combined into eight 2μL multiplex PCR sets. Each 2μL PCR plex contained 2–5 primer sets (each set at a concentration of 0.2μM and a total volume of 1μl), 1μl of Qiagen HotStar Multiplex PCR Master Mix and <10ng of lyophilised DNA (Kenta *et al*. 2008). PCR amplification was performed using a DNA Engine Tetrad PTC-225 thermal cycler (MJ Research, Bio-Rad, UK).

We used the following PCR program: initial denaturation at 95°C for 15 min, followed by 35 cycles of 94°C for 30s, 57°C for 90s and 72°C for 60s, followed by a final extension at 60°C for 30 min. PCR products were diluted with H_2_O (1 in 300) and loaded on to a ABI 3730 48-well DNA Sequencer (dye set DS-30, filter set D, and LIZ size standard). Allele sizes were assigned using the GeneMapper software (Applied Biosystems, USA).

We used the software Micro-Checker v2.2.3 (Van Oosterhout *et al*. 2004) to test for allelic dropout, scoring errors and the presence of null alleles. To estimate scoring errors, we repeated 15% of all genotyping reactions and found an overall scoring error rate of 0.3% per locus. Loci were tested if under selection with the software Lositan (Antao *et al*. 2008), using 50,000 simulations and the Stepwise Mutation Model.

The genotyping of individuals of known sex (87 females and 45 males) allowed us to rule out the possibility of sex-linkage. Observed and expected heterozygosities, and estimated null allele frequencies were calculated using CERVUS v3.0.3 (Kalinowski *et al.* 2007) and Micro-Checker (Van Oosterhout *et al*. 2004). Tests for departures from Hardy-Weinberg equilibrium and assessment of linkage disequilibrium were conducted on locations with more than five individuals in GENEPOP v4.0.10 (Raymond & Rousset 1995; Rousset 2008).

Out of the 39 loci, we identified a set of 23 autosomal polymorphic microsatellite markers that were in Hardy-Weinberg equilibrium in at least 14 out of the 16 colonies (locations with more than five samples) and had low estimated frequency of null alleles (Table S2). Additionally, these loci were neither under selection nor in linkage disequilibrium.

## Genetic Data Analysis

Bayesian phylogenetic trees were ran in MrBayes v3.2.1 (Ronquist & Huelsenbeck 2003) with the Generalized Time Reversible (GTR) model of DNA substitution with proportion of invariable sites model of rate variation (selected by jModelTest v0.1.1 [Posada 2008] based on AIC scores). We ran 6 × 10^7^ generations with four chains, sampled every 50^th^ generation, and two simultaneous runs, discarding the first 25% of trees generated as burn-in. Trees and posterior probabilities were visualized with Figtree v1.3.1 (<http://tree.bio.ed.ac.uk/software/figtree/>). Haplotype networks were constructed with Network ([www.fluxus-engineering.com](http://www.fluxus-engineering.com/) ) employing the median-joining network algorithm (Bandelt *et al*. 1999) and the Greedy FHP (‘prior to further processing’) distance calculation method.

Analysis of mtDNA and microsatellite genetic diversity was carried out at the geographical population level. MtDNA nucleotide polymorphism, haplotype diversity and genetic differentiation between regions were calculated using the software DnaSP v5.10 (Librado & Rozas 2009), with 10^4^ permutations to obtain probability values. Analysis of microsatellite genetic diversity, including allele frequencies, presence of private alleles, allelic richness, and gene diversity, was carried out with the programs FSTAT v2.9.3.2 (Goudet 1995) and GenAlEx v6.4 (Peakall & Smouse 2006) controlling for differences in sample size.

Analyzing STRUCTURE results: The number of distinct clusters was determined using STRUCTURE HARVESTER (Earl & Von Holdt 2012) based on the K value (number of clusters) at which the mean log-likelihood of observing the data under each K peaked or began to plateau, and where variation among runs was minimal (Figure S1_A_). Cluster assignment of individuals across replicate runs was aligned in CLUMPP (Jakobsson & Rosenberg 2007) using the Large K Greedy algorithm, and aligned assignments were visualized with the program DISTRUCT (Rosenberg 2004).

### Estimating sampling completeness

We assessed the accuracy of our estimate of haplotype diversity (haplotype sampling completeness) across *P. austriacus* range using sampling site-based Species Accumulation Curves and examining the resampling curve to determine whether it reached saturation (asymptote) within the available number of sampling sites. Species Accumulation Curves were performed at the *Cyt* *b* haplotype level with 95% confidence intervals with the program EstimateS v9.1.0 (Colwell 2013) using the default setting and 1000 randomisations, following the methods described in Pfenninger *et al*. (2012).

## Ecological Niche Modelling Procedures

Models were generated with 142 location records from across the species’ range that were confirmed genetically, either in this or previous studies (Juste *et al*. 2004; Spitzenberger *et al*. 2006; Ashrafi *et al*. 2010), including 32 location records from Iberia, 32 from the Balkans, nine from Italy, 13 from England, three from the Channel Isles and 53 from across Europe (ranging from France to Poland). We eliminated spatial autocorrelation between location records, using the Average Nearest Neighbor tool in ArcGIS v10 (ESRI) to remove duplicate and clustered location records. Multicollinearity between environmental variables was tested with ENMtools v1.3 (Warren *et al*. 2010).

Future models for 2080 were generated using the IPCC A2 scenario of continued population growth, medium economic growth and high reliance on fossil fuels (Nakicenovic *et al*. 2000). Because recent increases in emission rates have exceeded all scenario estimations it is recommend to use the more extreme ‘A’ scenarios rather than the more conservative ‘B’ scenarios (Beaumont *et al*. 2008).

The mean of the 50 runs was used to generate output maps, which are presented either as a continuous scale of suitability (0–1) or converted into binary maps of presence (1) / absence (zero). The 10^th^ percentile of training presence was used as the threshold value above which the species was assumed to be present (Phillips *et al*. 2009). Consensus maps of all models per time period (LGM and 2080) were created by averaging the output of the continuous maps or multiplying the binary maps. Stable areas, where the species is predicted to be present across time periods, were determined by overlaying maps across different time periods. All maps were manipulated in ArcGIS v10 (ESRI) using the raster calculator and reclassify tools.

## ABC Framework

### Specific model parameters and model checking procedure

Microsatellite loci were assumed to follow a Generalized Stepwise Mutation model (GSM). A uniform prior was assumed for the mean microsatellite mutation rate bounded between 10^-3^ and 10^-4^, a commonly used value in demographic models of bats (Storz & Beaumont 2002) and other taxa (Balloux & Lugon-Moulin 2002). Other microsatellite mutation model parameters were set to program default, allowing single nucleotide mutations. MtDNA mutation model parameters were set based on the results of jModelTest to follow the HKY mutation model. We assumed a uniform prior for the mean mtDNA (Cyt *b*) mutation rate bounded between 10^-8^and 10^-7^ (the Cyt *b* mutation rate range estimated in most studied bat species [Nabholz *et al.* 2008]).

Demographic prior parameters were defined based on prior knowledge of the species’ biology, population abundance estimates and timing of major climatic events. Uniform priors were assumed for all demographic parameters. Effective population size (Ne) priors were bounded between 10^3^ and 10^6^ for Iberian, Italian and Balkan populations, between 10^4^ and 10^6^ for the Western European population, and between 10^1^ and 10^4^ for the UK population due to its small estimated census population size (Harris *et al*. 1995). Population divergence time priors were bounded between 10^2^ and 10^5^ generations. Priors for admixture rates were bounded between 0.01 and 0.99.

We used 70–96 summary statistics per analysis set. For the microsatellite loci we used three single sample statistics (mean number of alleles, mean Nei’s genetic diversity index and mean allele size variance), and three between-sample statistics (Fst, mean allele size variance, and mean index of classification). For the mtDNA sequence we used four single sample statistics (number of distinct haplotypes, number of segregating sites, mean pairwise differences, and Tajima’s D statistics) and three between-sample statistics (Fst and mean within and between sample pairwise differences).

During the model checking stage we simulated 10^3^ pseudo-observed data sets from the posterior predictive distribution of parameters, ranking each summary statistic against those obtained from the observed data set to test whether replicated data fell outside the probability distribution of the observed data. Bonferroni corrections were applied to account for multiple testing when necessary. To avoid over-estimating model fit (Cornuet *et al*. 2010), we used at this stage a different set of between-sample summary statistics from the ones used originally to compute the posterior distribution of parameters (for microsatellite loci: mean number of alleles across loci, mean gene diversity across loci and shared allele distance; for mtDNA sequence: number of distinct haplotypes, number of segregating sites and mean of within sample pairwise differences).

As suggested by Robert *et al*. (2011) and Estoup *et al*. (2012) we evaluated the power of the model to discriminate among scenarios (confidence in scenario choice). We evaluated model specificity (type 1 error) by simulating 500 pseudo-observed datasets with the ABC chosen scenario and calculating rates of false scenario assignment (highest posterior probabilities for the wrong scenarios). Model sensitivity (type 2 error) was calculated based on the proportion of 500 pseudo-observed datasets simulated with other scenarios that were assigned to the scenario selected by the ABC analysis (Cornuet *et al*. 2010).

### ABC Analysis 1

The first analysis aimed to identify the source *P. austriacus* population among the three locations identified by palaeo-ENMs as having suitable climatic conditions during glaciation periods (Iberia, Italy and the Balkans), and patterns of range expansion. The first scenario (Scenario 1.1) considers the presence of a single source population in Iberia from where the rest of the range was colonized either directly (Italy and Western Europe) or via Western Europe (Balkans). Scenarios 1.2 and 1.4 considered two source populations, Iberia and Italy, and subsequent colonization of Western Europe either through an admixture of Iberian and Italian populations (1.2) or from Iberia alone (1.4). Scenarios 1.3 and 1.5 compared three source populations (Iberia, Italy and Balkans) and subsequent range expansion either through admixture between Iberian and Balkan populations (1.3) or from the Balkans alone (1.5) (Figure S6_A_).

### ABC Analysis 2

The second analysis included the potential of range colonization from an unsampled refugial population from southern France (a location identified as having suitable conditions during the LGM). A preliminary analysis was run to determine the pattern of colonization of the northern edge of the range (England), comparing models of long-range colonization directly from Iberia (Scenario P1), admixture between Western European (France, Belgium and Germany) and Iberian populations to create the English population (Scenario P2) and stepping stone colonization from Iberia to Western Europe and then England (Scenario P3). The data supported a stepping stone model of northern edge-of-range recolonisation from Iberia to Western Europe and from Western Europe to England (Fig. S7), and therefore in the second analysis range colonization from glacial refugia to the northern edge of the range was set to follow a stepping stone pattern.

Scenario 2.1 was equivalent to scenario 1.1 in the first analysis, considering a single refugium in Iberia from where the rest of the range was colonized in a stepping stone manner. Scenario 2.2 considered two refugial populations, Iberia and the unsampled population, with the unsampled population colonizing the rest of the range. Scenarios 2.3 and 2.4 considered three refugial populations, Iberia, the unsampled population and either the Balkans (2.3) or Italy (2.4). In both cases the range was recolonized through an admixture between the unsampled population and either the Balkan or Italian population. The final scenario (2.5) compared two source populations (Iberia and Italy) and range colonization from Italy only (Figure S6_B_).

### ABC Analysis 3

The final analysis examined the demographic history of the English edge-of-range population and changes in population size since colonisation. We compared a null model of no change in population size (Scenario 3.1) to a model of a short bottleneck (10 generations) during colonisation followed by population expansion (Scenario 3.2) and a model of recent (1–10^3^ generations) change (increase or decrease) in population size (Scenario 3.3) (Fig. S8)

## References

Ashrafi, S., Bontadina, F., Kiefer, A., Pavlinic, I. & Arlettaz R. (2010). Multiple morphological characters needed for field identification of cryptic long-eared bat species around the Swiss Alps. *J. Zool.,* 281, 241–248.

Alfadala, S., Dawson, D.A., Horsburgh, G.J., Behnke, J.M., Bajer, A., Mohallal, E.M.E. *et al.* (2012). Large-scale isolation of Eastern spiny mouse *Acomys dimidiatus* microsatellite loci through GS-FLX 454 titanium sequencing. *Conserv. Gen. Res*., DOI 10.1007/s12686-012-9842-z.

Antao, T., Lopes, A., Lopes, R.J,. Beja-Pereira, A. & Luikart, G. (2008). LOSITAN: a workbench to detect molecular adaptation based on a Fst-outlier method. *BMC Bioinformatics*, 9, 323.

Balloux, F. & Lugon-Moulin, N. (2002). The estimation of population differentiation with microsatellite markers. *Mol. Ecol*., 11, 155–165.

Bandelt, H-J., Forster, P. & Röhl, A. (1999). Median-joining networks for inferring intraspecific phylogenies. *Mol. Biol. Evol*., 16, 37–48.

Beaumont, L.J., Hughes, L. & Pitman, A.J. (2008). Why is the choice of future climate scenarios for species distribution modelling important? *Ecol. Lett.,* 11, 1135–1146.

Burland, T.M., Barratt, E.M. & Racey, P.A. (1998). Isolation and characterization of microsatellite loci in the brown long-eared bat, *Plecotus auritus*, and crossspecies amplification within the family Vespertilionidae. *Mol. Ecol*., 7, 136–138.

Colwell, R.K. (2013). Estimates: statistical estimation of species richness and shared species from samples, version 9. User’s guide and application published at <http://viceroy.eeb.uconn.edu/estimates/> (17 June 2013, date last accessed).

Cornuet, J.M., **Ravigné, V. & Estoup, A. (2010).** Inference on population history and model checking using DNA sequence and microsatellite data with the software DIYABC (v1.0). *BMC Bioinformatics,* 11, 401.

Earl, D.A. & Von Holdt, B.M. (2012). STRUCTURE HARVESTER: a website and program for visualizing STRUCTURE output and implementing the Evanno method. *Conserv. Gen. Res*., 4, 359–361.

Edgar, R.C. (2004). MUSCLE: multiple sequence alignment with high accuracy and high throughput. *Nucleic Acids Res*., 32, 1792–1797.

Estoup, A., Lombaert, E., Marin, J.M., Guillemaud, T., Pudlo, P., Robert, C.P. *et al.* (2012). Estimation of demo-genetic model probabilities with Approximate Bayesian Computation using linear discriminant analysis on summary statistics. *Mol. Ecol. Res*., 12, 846–855.

Goudet, J. (1995). FSTAT (Version 1.2): A computer program to calculate F-statistics. *J. Hered.,* 86, 485–486.

Harris, S., Morris, P., Wray, S. & Yalden, D. (1995) A Review of British Mammals: Population Estimates and Conservation Status of British Mammals other than Cetaceans. JNCC, Peterborough, UK.

Jakobsson, M. & Rosenberg, N. (2007). CLUMPP: a cluster matching and permutation program for dealing with label switching and multimodality in analysis of population structure. *Bioinformatics*, 23, 1801–1806.

Juste, J., Ibáñez, C., Munoz, J., Trujillo, D., Benda, P., Karatas, A. *et al.* (2004). Mitochondrial phylogeography of the long-eared bats (*Plecotus*) in the Mediterranean Palaearctic and Atlantic Islands. *Mol. Phylogenet. Evol*., 31, 1114–1126.

Kalinowski, S.T., Taper, M.L. & Marshall, T.C. (2007). Revising how the computer program CERVUS accommodates genotyping error increases success in paternity assignment. *Mol. Ecol*., 16, 1099–1006.

Kenta, T., Gratten, J., Haigh, N.S., Hinten, G.N., Slate, J., Butlin, R.K. *et al.* (2008). Multiplex SNP-SCALE: a cost-effective medium-throughput SNP genotyping method. *Mol. Ecol. Res*., 8, 1230–1238.

Librado, P. & Rozas, J. (2009). DnaSP v5: A software for comprehensive analysis of DNA polymorphism data. *Bioinformatics*, 25, 1451–1452.

Nabholz, B., Glemin, S. & Galtier, N. (2008). Strong variations of mitochondrial mutation rate across mammals-the longevity hypothesis. *Mol. Biol. Evol*., 25, 120–130.

Nakicenovic, N., Alcamo, J., Davis, G., de Vries, B., Fenhann, J., Gaffin, S. *et al*. (2000). *Special report on emissions scenarios: a special report of Working Group III of the Intergovernmental Panel on Climate Change*. Cambridge University Press, NY, USA.

Peakall, R. & Smouse, P.E. (2006). GENALEX 6: genetic analysis in Excel. Population genetic software for teaching and research. *Mol. Ecol. Notes,* 6, 288–295.

Pfenninger, M., Bálint, M. & Pauls, S.U. (2012). Methodological framework for projecting the potential loss of intraspecific genetic diversity due to global climate change. *BMC Evol. Biol.,* 12, 224.

Phillips, S.J., Dudik, M., Elith, J., Graham, C.H., Lehmann, A., Leathwick, J*. et al.* (2009). Sample selection bias and presence-only distribution models: implications for background and pseudo-absence data. *Ecol. Applications,* 19, 181–197.

Posada, D. (2008). jModelTest: Phylogenetic Model Averaging, *Mol. Biol. Evol*., 25, 1253–1256.

Raymond, M. & Rousset, F. (1995). GENEPOP (version 1.2): population genetics software for exact tests and ecumenicism. *J. Hered*., 86, 248–249.

Robert, C.P., Cornuet, J.M., Marin, J.M. & Pillai, N.S. (2011). Lack of confidence in approximate Bayesian computation model choice. *Proc. Natl. Acad. Sci. USA,* 108, 15112–15117.

Ronquist, F. & Huelsenbeck, J.P. (2003) .MrBayes 3: Bayesian phylogenetic inference under mixed models. *Bioinformatics*, 19, 1572–1574.

Rosenberg, N.A. (2004). DISTRUCT: a program for the graphical display of population structure. *Mol. Ecol. Notes,* 4, 137–138.

Rousset, F. (2008). GENEPOP'007: a complete re-implementation of the GENEPOP software for Windows and Linux. *Mol. Ecol. Res*., 8, 103–106.

Rozen, S. & Skaletsky, H.J. (2000). Primer3 on the WWW for general users and for biologist programmers. *In Bioinformatics Methods and Protocols: Methods in Molecular Biology* (eds. Krawetz, S. & Misener, S.). Humana Press, USA, pp. 365–386.

Rutishauser, M. D., Bontadina, F., Braunisch, V., Ashrafi, S., Arlettaz, R. (2012). The challenge posed by newly discovered cryptic species: disentangling the environmental niche of long-eared bats. *Diversity Distrib*., 18, 1107–1119.

Spitzenberger, F., Strelkov, P., Winkler, H. & Haring, E. (2006). A preliminary revision of the genus *Plecotus* (Chiroptera, Vespertilionidae) based on genetic and morphological results. *Zool. Scr.,* 35, 187–230.

Storz, J.F. & Beaumont, M. (2002). Testing for genetic evidence of population expansion and contraction: an empirical analysis of microsatellite DNA variation using a hierarchical Bayesian model. *Evol*., 56, 154–166.

Van Oosterhout, C., Hutchinson, W.F., Wills, D.P.M. & Shipley, P. (2004). MICRO-CHECKER: software for identifying and correcting genotyping errors in microsatellite data. *Mol. Ecol. Notes,* 4, 535–538.

Warren, D.L., Glor, R.E. & Turelli, M. (2010). ENMTools: a toolbox for comparative studies of environmental niche models. *Ecography*, 33, 607–611.

Xia, X. & Xie, Z. (2001). DAMBE: Data analysis in molecular biology and evolution. *J. Hered.*, 92, 371–373.
