# Supplementary material for: The shaping of genetic variation in edge-of-range populations under past and future climate change
Source: Ecol Lett. 2013 Jul 26;16(10):1258–66. doi: 10.1111/ele.12158 (PMC4015367; doi:10.1111/ele.12158)
Supplement: Supplementary file 2 [file ele0016-1258-sd2.doc]

# Supporting Tables

**Table S1** – The geographic location (WGS1984 co-ordinates) of the 259 *P. austriacus* genetic samples included in the study, with the name of sample collector and the number of samples collected from each location. Regions correspond to the main geographical areas used in the ABC inference.

| **Region** | **Country** | **Site** | **Location** | **N** | **Collector / sample provider** |
| --- | --- | --- | --- | --- | --- |
| England | England | West Devon | 50.6, -3.6 | 17 | OR |
|  |  | East Devon | 50.8, -3.0 | 3 | OR |
|  |  | Somerset | 51.1, -2.7 | 1 | VLA |
|  |  | Dorset1 | 50.9, -2.3 | 2 | Colin Morris |
|  |  | Dorset2 | 50.6, -2.3 | 9 | OR |
|  |  | Dorset3 | 50.7, -2.1 | 1 | OR |
|  |  | New Forest | 50.8, -1.7 | 1 | David Fisher |
|  |  | Isle of Wight W | 50.6, -1.3 | 10 | OR |
|  |  | Isle of Wight E | 50.7, -1.1 | 7 | OR |
|  |  | Sussex | 51.0, -0.6 | 3 | OR, Daniel Whitby |
| Channel Isles | Guernsey |  | 49.4, -2.6 | 12 | Pat Costen, OR, VLA |
|  | Jersey | Jersey E | 49.2, -2.0 | 8 | OR, Hugh Forshaw |
|  |  | Jersey W | 49.2, -2.2 | 4 | OR, Hugh Forshaw, VLA |
| Western Europe | France | Haute-Garonne | 43.7, 1.4 | 1 | AK |
|  |  | Dordogne | 44.6, 1.1 | 1 | AK |
|  |  | Charente | 45.4, 0.1 | 1 | Bourges Museum |
|  |  | Cher1 | 46.7, 2.1 | 2 | Bourges Museum |
|  |  | Cher2 | 47.0, 3.0 | 1 | AK |
|  |  | Cher3 | 47.1, 2.4 | 4 | Bourges Museum, AK |
|  |  | Cher4 | 47.2, 2.1 | 3 | Bourges Museum, AK |
|  |  | Loire-Atlantique1 | 47.3, -1.9 | 10 | SP |
|  |  | Loire-Atlantique2 | 47.4, -2.3 | 1 | Bourges Museum |
|  |  | Morbihan | 47.6, -2.7 | 1 | Bourges Museum |
|  |  | Loir et Cher | 47.9, 0.9 | 1 | Bourges Museum |
|  |  | Moselle | 49.0, 6.9 | 1 | Bourges Museum |
|  |  | Marne | 49.1, 3.8 | 1 | AK |
|  |  | Pas de Calais | 50.2, 2.6 | 1 | AK |
|  | Luxembourg |  | 50.0, 5.1 | 1 | Gregory Motte |
|  | Belgium | Hainaut | 50.3, 4.4 | 1 | Gregory Motte |
|  |  | Liege | 50.7, 5.7 | 1 | Gregory Motte |
|  | Germany | Sulzbach | 49.3, 7.1 | 1 | AK |
|  |  | Fischbach/Nahe | 49.6, 7.2 | 2 | AK |
|  |  | Rheinland-Pfalz1 | 49.8, 7.8 | 3 | AK |
|  |  | Rheinland-Pfalz2 | 50.3, 7.3 | 7 | AK |
|  | Switzerland | Mandach | 47.5, 8.2 | 12 | RA* |
|  | Austria^†^ |  | 47.1, 15.8 | 2 | AK |
| Balkans | Greece | Meteora | 39.7, 21.6 | 1 | AK |
|  |  | Florina | 40.8, 21.4 | 1 | AK |
|  | Albania |  | 41.2, 20.2 | 1 | AK |
|  | Bulgaria | Oreshari | 41.6, 24.4 | 1 | AK |
|  |  | Burgos | 42.6, 27.4 | 1 | AK |
|  |  | Sofia | 42.7, 23.2 | 2 | AK |
|  |  | Sliven | 42.7, 26.3 | 1 | AK |
|  |  | Pleven | 43.6, 24.9 | 2 | AK |
|  | Croatia |  | 44.7, 14.9 | 1 | AK |
|  | Hungary |  | 47.5, 19.0 | 2 | AK |
|  | Slovakia |  | 48.6, 20.9 | 1 | AK |
| Italy | Italy | Toscana1 | 43.8, 11.3 | 6 | Zoological Museum Florence |
|  |  | Toscana2 | 43.9, 10.9 | 5 | Zoological Museum Florence |
|  | Sardinia |  | 39.5, 8.9 | 3 | AK |
|  | Corsica |  | 42.5, 9.2 | 2 | AK, Bourges Museum |
| Iberia | Spain | Granada | 37.1, -3.4 | 12 | CI, JJ |
|  |  | Almería | 37.3, -2.5 | 18 | CI, JJ |
|  |  | Jaén | 37.9, -2.9 | 14 | CI, JJ |
|  |  | La Rioja | 42.2, -2.6 | 14 | CI, JJ |
|  |  | Málaga | 36.7, -4.9 | 1 | CI, JJ |
|  |  | Huelva1 | 37.6, -6.7 | 1 | CI, JJ |
|  |  | Huelva2 | 37.9, -6.8 | 1 | CI, JJ |
|  |  | Sevilla | 38.0, -6.2 | 1 | CI, JJ |
|  |  | Córdoba | 38.0, -4.8 | 1 | CI, JJ |
|  |  | Cáceres | 39.9, -6.3 | 1 | CI, JJ |
|  |  | Minorca | 39.9, 4.3 | 1 | CI, JJ |
|  |  | Ávila | 40.3, -5.3 | 1 | CI, JJ |
|  |  | Tarragona | 41.4, 1.1 | 1 | CI, JJ |
|  |  | Huesca1 | 41.6, 0.0 | 1 | CI, JJ |
|  |  | Huesca2 | 42.6, 0.0 | 1 | CI, JJ |
|  |  | Zaragoza | 41.7, -1.7 | 1 | CI, JJ |
|  |  | Soria | 42.0, -2.7 | 1 | CI, JJ |
|  |  | Navarra | 42.1, -1.6 | 1 | CI, JJ |
|  |  | Pontevedra | 42.2, -8.8 | 1 | CI, JJ |
|  |  | Girona | 42.3, 2.6 | 1 | CI, JJ |
|  |  | Orense | 42.4, -7.5 | 1 | CI, JJ |
|  |  | Lugo | 42.5, -7.3 | 1 | CI, JJ |
|  |  | A Coruña | 43.7, -8.0 | 1 | CI, JJ |
|  | Portugal | Aqueducto | 38.8, -9.2 | 6 | HR |
|  |  | Monserrate | 38.8, -9.4 | 1 | HR |
|  |  | Caramulo | 40.6, -8.2 | 1 | HR |
|  |  | Reboredo | 41.2, -7.0 | 1 | HR |
|  |  | Canaveses | 41.2, -8.2 | 1 | HR |
|  |  | Picote | 41.4, -6.4 | 1 | HR |
|  |  | Carrazedo | 41.6, -8.4 | 2 | HR |

* Rutishauser *et al.* (2012 [reference in Appendix S1])

† Not included in ABC models because it is located between the Balkans and Mainland Western Europe

**Table S2** – Microsatellite loci and primer sequences used in the study, including information on motif, product size, observed (H_obs_) and expected (H_exp_) heterozygosity, P values for deviation from Hardy-Weinberg equilibrium (P(HW), based on sampling locations with more than five individuals), and estimated frequency of null alleles. (EMBL accession numbers HE983997–HE984016)

| **Primer name** | **Repeat motif** | **Sequence (5’-3’)** | **Allele size range** | **Number of alleles** | **H_obs_** | **H_exp_** | **P (HW)** | **F (Null alleles)** |
| --- | --- | --- | --- | --- | --- | --- | --- | --- |
| *Paust01* | TATC | F: [HEX] TCATTGACCAAGAAATTACTAGTTAGG | 184-208 | 7 | 0.67 | 0.74 | 0.44 | 0.05 |
|  |  | R: AGCCAAATTGGTAGGAAACC |  |  |  |  |  |  |
| *Paust02* | CA | F: [6-FAM] CATTTCATGGGTCCTGTTCC | 185-214 | 13 | 0.82 | 0.85 | 0.93 | 0.02 |
|  |  | R:TGCTAATTGACTATTCTCATCTTTGG |  |  |  |  |  |  |
| *Paust03* | GA | F: [HEX] GGCTTTCCAATAGAACATCCTTC | 246-257 | 5 | 0.51 | 0.56 | 0.95 | 0.05 |
|  |  | R: GGAGGCAACCAATCATCG |  |  |  |  |  |  |
| *Paust04* | TTTC | F: [HEX] GTCCTCCCAAGCTCACCTG | 256-309 | 9 | 0.62 | 0.73 | 0.63 | 0.08 |
|  |  | R: ATGGAGGAGGTAACCCATCG |  |  |  |  |  |  |
| *Paust05* | CA | F: [HEX ]CCAGAGTGGGAAGTAAATCTGC | 234-263 | 14 | 0.79 | 0.80 | 0.84 | 0.01 |
|  |  | R: TGTTTATCAGCATGAAGGCATTAG |  |  |  |  |  |  |
| *Paust06* | CA (GT) | F: [6-FAM] AAGGAAGGACAACTGGAACAG | 131-158 | 12 | 0.82 | 0.83 | 0.78 | 0.00 |
|  |  | R: AACTGAATAGACAACTCAGATTCTCC |  |  |  |  |  |  |
| *Paust07* | CA | F: [6-FAM] GGCTCCTGCACTGTGACC | 212-245 | 14 | 0.83 | 0.87 | 0.81 | 0.02 |
|  |  | R: GACTGCAGCCAACACGAAG |  |  |  |  |  |  |
| *Paust08* | GT | F: [HEX] TTGCAATGGTTCTGATGAGG | 147-171 | 6 | 0.44 | 0.48 | 0.99 | 0.04 |
|  |  | R: TAGCTTCCCTGTCCATCCAC |  |  |  |  |  |  |
| *Paust09* | CA | F: [6-FAM] GGTGGGAATGAAACATCCAC | 260-262 | 2 | 0.31 | 0.39 | 0.71 | 0.11 |
|  |  | R: AAATTTGGGAGGAAGCAAGG |  |  |  |  |  |  |
| *Paust10* | GT | F: [HEX] CCCTCTACCCATTCAACAAGC | 94-135 | 19 | 0.81 | 0.91 | 0.56 | 0.05 |
|  |  | R: CGCTGATGTTACAACTCATTGTG |  |  |  |  |  |  |
| *Paust11* | CA | F: [6-FAM] TCATTGCAACATTATTTATAGTAGCC | 175-187 | 6 | 0.32 | 0.43 | 0.09 | 0.18* |
|  |  | R: ACCCTCAAAGTTTATCCATG |  |  |  |  |  |  |
| *Paust12* | GATA | F: [6-FAM] AGCGAGCAATCAAACTCCTG | 134-174 | 13 | 0.73 | 0.76 | 0.32 | 0.02 |
|  |  | R: AATCAGGATGCAGCTGGAAG |  |  |  |  |  |  |
| *Paust13* | GATA | F: [6-FAM] TGAGCCAATACCTTATAACAAACC | 172-199 | 10 | 0.73 | 0.78 | 0.75 | 0.03 |
|  |  | R: GCAAGATAGAATATGGGCACTG |  |  |  |  |  |  |
| *Paust14* | GATA | F: [6-FAM] AGTGACCAGTCATTCCAGTCG | 145-181 | 11 | 0.62 | 0.64 | 0.91 | 0.01 |
|  |  | R: CACTCCAGGCCTTCACTACC |  |  |  |  |  |  |
| *Paust15* | GT | F: [HEX] GATGAAGAGTCCATGGTGTTCTG | 101-134 | 17 | 0.85 | 0.89 | 0.88 | 0.02 |
|  |  | R: TACCCTCGCTCTGAGGACTG |  |  |  |  |  |  |
| *Paust16* | CA | F: [HEX] AAGTGGGACTGGAGCTGGTC | 133-187 | 15 | 0.81 | 0.86 | 0.62 | 0.03 |
|  |  | R: GGGTGCTTGGTGACACTGAC |  |  |  |  |  |  |
| *Paust17* | GT | F: [HEX] TTGGCAGTCTCTATCCCAAG | 162-185 | 7 | 0.53 | 0.58 | 0.99 | 0.05 |
|  |  | R: ACAGTCAGCCAGGAAATCAC |  |  |  |  |  |  |
| *Paust18* | CA | F: [HEX] TGGAATAGTTCCATGTCTTGACTC | 134-157 | 12 | 0.81 | 0.84 | 0.85 | 0.02 |
|  |  | R: GCAGTGCTTAACTCAGGAACAG |  |  |  |  |  |  |
| *Paust19* | GT | F: [HEX] TGAATGCAGGCCTAACTGAC | 184-226 | 15 | 0.81 | 0.87 | 0.88 | 0.03 |
|  |  | R: CACATAAGAATCAACCAATAGATGC |  |  |  |  |  |  |
| *Paust20* | GT | F: [6-FAM] CGGAGACTGGAGGAGTACG | 98-122 | 10 | 0.51 | 0.71 | 0.02^†^ | 0.16 |
|  |  | R: AATTCGCAGCTGTCCTACC |  |  |  |  |  |  |
| *Paur01*^‡^ | GT | F: [HEX] CAATTTCAAGGCAGTGCTC | 132-159 | 12 | 0.74 | 0.80 | 0.98 | 0.04 |
|  |  | R: TGCTGTCCCTGCATGCTG |  |  |  |  |  |  |
| *Paur05*^‡^ | GT | F: [HEX] GGACAGTATGCCATGTTATGCTG | 229-249 | 11 | 0.75 | 0.79 | 0.57 | 0.03 |
|  |  | R: GCACTTTCACAAACCTAGATGG |  |  |  |  |  |  |
| *Paur06*^‡^ | AC (AG) | F: [6-FAM] GATCAGATTTCCAAACAGAG | 143-169 | 10 | 0.75 | 0.81 | 0.94 | 0.04 |
|  |  | R: AGGTTCTTTCTTCAGCTATG |  |  |  |  |  |  |

*Two out of the 16 locations with more than five samples had estimated null alleles at this locus.

† Deviation from Hardy-Weinberg equilibrium (P<0.05 no correction for multiple tests applied) in two out of the 16 locations with more than five samples

‡These three loci were published by Burland *et al*. (1998 [reference in Appendix S1]).

**Table S3** – Genetic differentiation between *P. austriacus* geographical populations with pairwise θ_ST_ estimates, based on Cyt *b* mtDNA sequences, above the diagonal line, and pairwise F_ST_ estimates, based on the 23 microsatellite loci, below the line. Significance values for θ_ST_ estimates based on Chi-Square tests are presented as: ** P<0.01, and *** P<0.001 (Channel – Channel Isles, Europe – Mainland Western Europe).

|  | **England** | **Channel** | **Europe** | **Italy** | **Iberia** | **Balkans** |
| --- | --- | --- | --- | --- | --- | --- |
| **England** |  | 0.057 | 0.033 | 0.624*** | 0.824*** | 0.017 |
| **Channel** | 0.036 |  | 0.000 | 0.636*** | 0.828*** | 0.000 |
| **Europe** | 0.034 | 0.036 |  | 0.626*** | 0.825*** | 0.000 |
| **Italy** | 0.064 | 0.067 | 0.030 |  | 0.702*** | 0.606** |
| **Iberia** | 0.050 | 0.046 | 0.025 | 0.035 |  | 0.819*** |
| **Balkans** | 0.068 | 0.089 | 0.034 | 0.059 | 0.067 |  |

**Table S4** – Estimated contemporary gene flow rates between the six geographical populations, showing rates of gene flow from populations in the horizontal rows into populations along the vertical columns. The proportion of bats remaining in their respective natal population is highlighted in bold (Channel – Channel Isles, Europe – Mainland Western Europe).

|  | **England** | **Channel** | **Europe** | **Italy** | **Iberia** | **Balkans** |
| --- | --- | --- | --- | --- | --- | --- |
| **England** | **0.972** | 0.005 | 0.007 | 0.005 | 0.006 | 0.005 |
| **Channel** | 0.257 | **0.680** | 0.024 | 0.013 | 0.014 | 0.012 |
| **Europe** | 0.174 | 0.024 | **0.783** | 0.005 | 0.028 | 0.005 |
| **Italy** | 0.019 | 0.017 | 0.236 | **0.685** | 0.027 | 0.017 |
| **Iberia** | 0.006 | 0.014 | 0.037 | 0.027 | **0.943** | 0.005 |
| **Balkans** | 0.026 | 0.019 | 0.228 | 0.020 | 0.020 | **0.687** |

**Table S5 – List of *Cyt* *b* *P. austriacus* haplotypes identified in this study, their GenBank accession numbers and the geographical location (WGS1984) of samples included in the haplotype (Europe = the pan-European common haplotype).**

| **Haplotype ID** | **GenBank Accession number** | **Location** |  |
| --- | --- | --- | --- |
| England | KF358491 | Dorset, England (50.6, -2.3) |  |
| France | KF358492 | Cher, France (47.2, 2.1) |  |
| Greece | KF358493 | Meteora, Greece (39.7, 21.6) |  |
| Italy | KF358494 | Toscana, Italy (43.8, 11.3; 43.9, 10.9) |  |
| Europe | KF358495 | Most English, Channel Isles, Western European and Balkan locations |  |
| Iberia01 | KF358496 | Ávila, Spain (40.3, -5.3) |  |
| Iberia02 | KF358497 | Baleares, Spain (39.9, 4.3) |  |
| Iberia03 | KF358498 | Cáceres, Spain (39.9, -6.3) |  |
| Iberia04 | KF358499 | Girona, Spain (42.4, 2.6) |  |
| Iberia05 | KF358500 | Huesca2, Spain (42.6, 0.0) |  |
| Iberia06 | KF358501 | Jaén, Spain (37.9, -2.9) |  |
| Iberia07 | KF358502 | Huesca1, Spain (41.6, 0.0) |  |
| Iberia08 | KF358503 | Málaga, Spain (36.7, -4.9) |  |
| Iberia09 | KF358504 | Navarra, Spain (42.1, -1.6) |  |
| Iberia10 | KF358505 | Orense, Spain (42.4, --7.5) |  |
| Iberia11 | KF358506 | Tarragona, Spain (41.4, 1.1) |  |
| Iberia12 | KF358507 | Zaragoza, Spain (41.7, -1.7) |  |
| Iberia13 | KF358508 | Reboredo Portugal (41.2, -7.0) |  |
| Iberia14 | KF358509 | Aqueducto, Portugal (38.8, -9.2) |  |
| Iberia15 | KF358510 | Canaveses, Portugal (41.2, -8.2) |  |
| Iberia16 | KF358511 | A Coruña, Spain (43.7, -8.0); Carrazedo, Portugal (41.6, -8.4) |  |
| Iberia17 | KF358512 | Huelva1, Spain (37.6, -6.7); Monserrate, Portugal (38.8, -9.4) |  |
| Iberia18 | KF358513 | Rioja, Spain (42.2, -2.6) |  |
| Iberia19 | KF358514 | Rioja, Spain (42.2, -2.6) |  |
| Iberia20 | KF358515 | Rioja, Spain (42.2, -2.6) |  |
| Iberia21 | KF358516 | Rioja, Spain (42.2, -2.6); Soria, Spain (42.0, -2.7) |  |
| Iberia22 | KF358517 | Jaén, Spain (37.9, -2.9) |  |
| Iberia23 | KF358518 | Jaén, Spain (37.9, -2.9) |  |
| Iberia24 | KF358519 | Jaén, Spain (37.9, -2.9) |  |
| Iberia25 | KF358520 | Jaén, Spain (37.9, -2.9) |  |
| Iberia26 | KF358521 | Almería, Spain (37.3, -2.5); Granada, Spain (37.1, -3.4);  Jaén, Spain (37.9, -2.9); Caramulo, Portugal (40.6, -8.2) |  |
| Iberia27 | KF358522 | Córdoba, Spain (38.0, -4.8); Picote, Portugal (41.4, -6.4); Rioja, Spain (42.2, -2.6) | |
