# Supplementary material for: The shaping of genetic variation in edge-of-range populations under past and future climate change
Source: Ecol Lett. 2013 Jul 26;16(10):1258–66. doi: 10.1111/ele.12158 (PMC4015367; doi:10.1111/ele.12158)

**Figure S1** – **Results of the STRUCTURE analysis**. A) Log-likelihood probability plots for dividing the whole dataset into 1–13 clusters, showing means and standard deviations of the ten replicate runs for each number of clusters. B) Patterns of cluster membership of individual samples, arranged based on their geographical location, for number of clusters ranging from K=2 (upper panel) to K=7 (bottom panel).


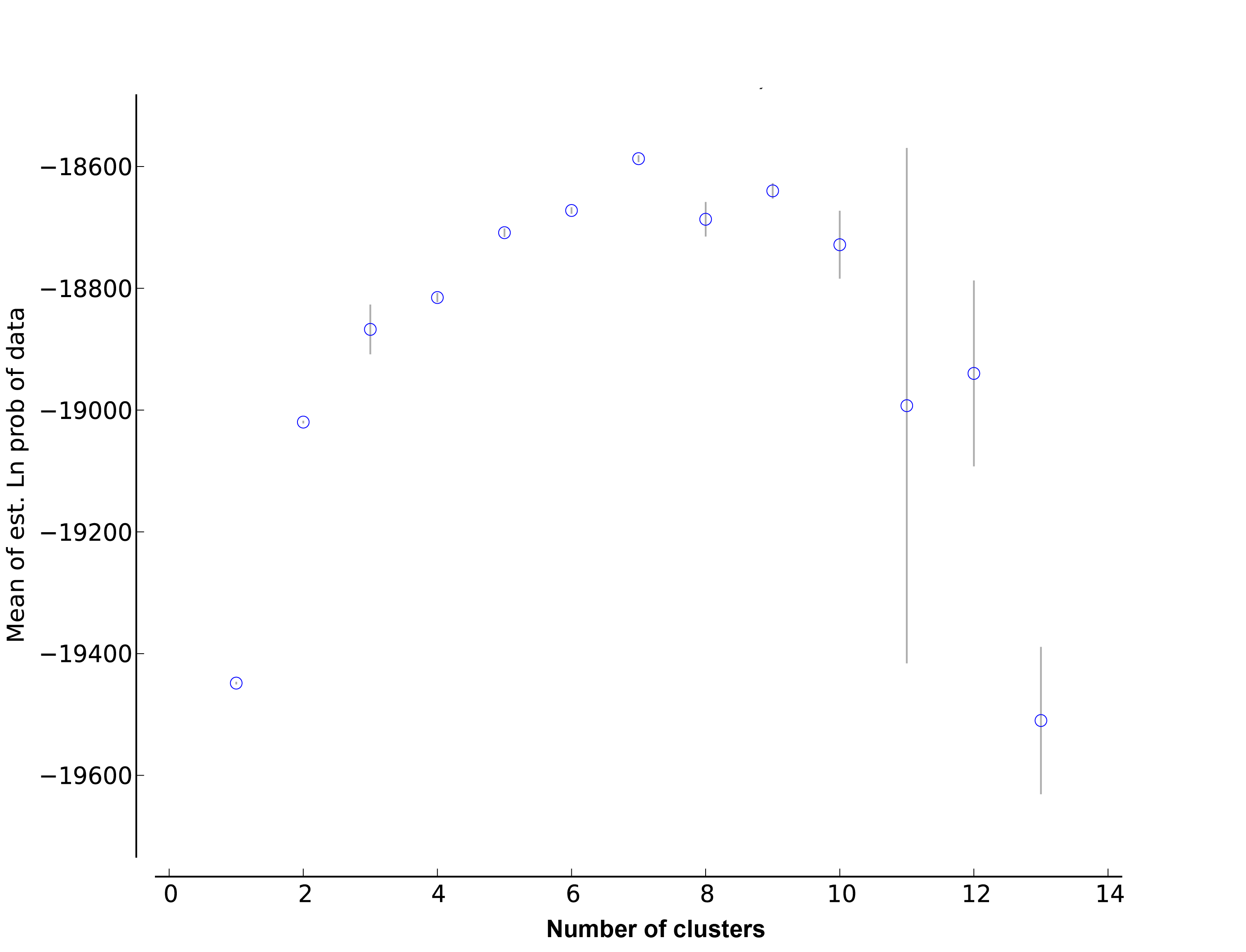


**A**

**B**


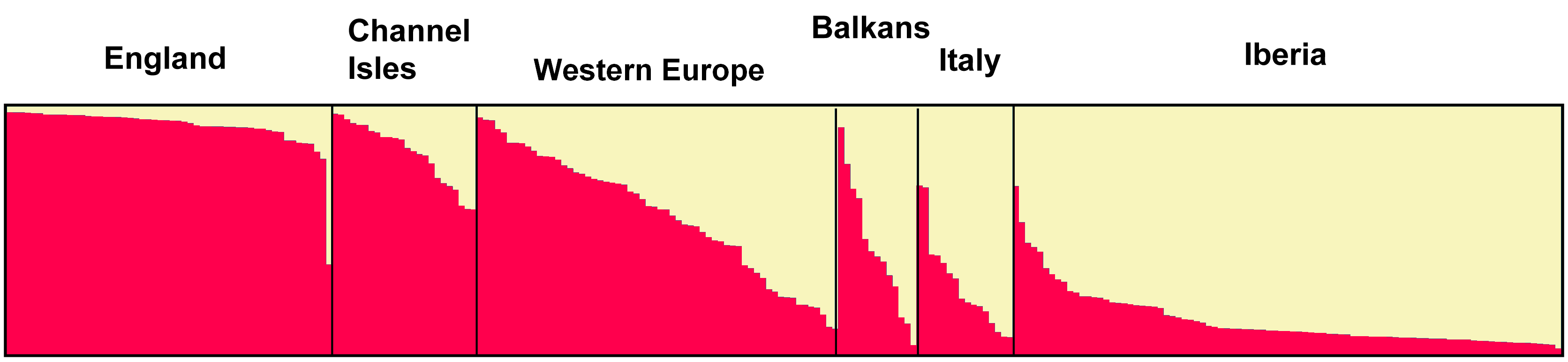


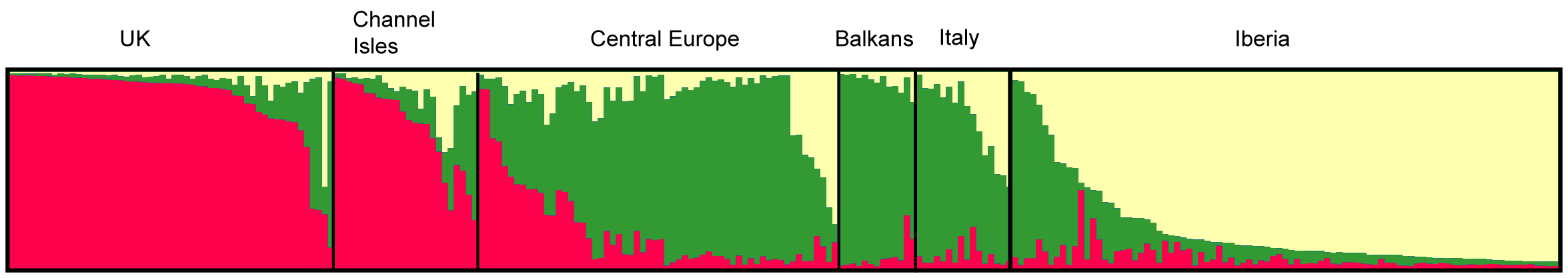


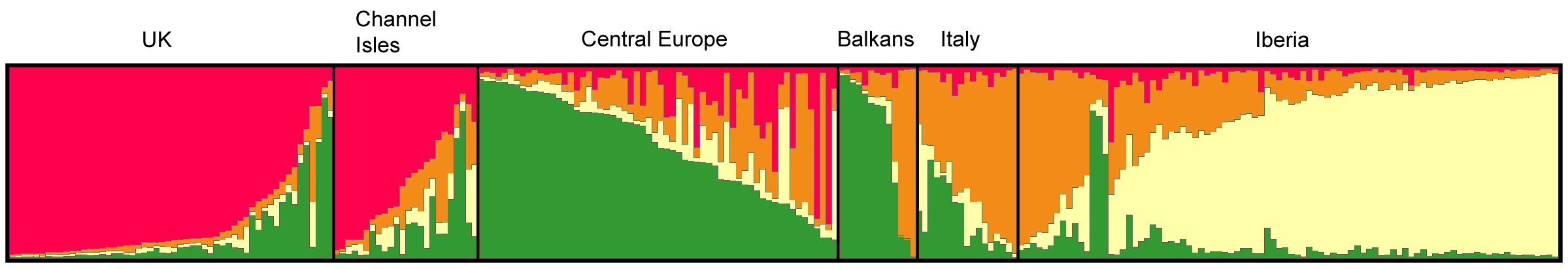


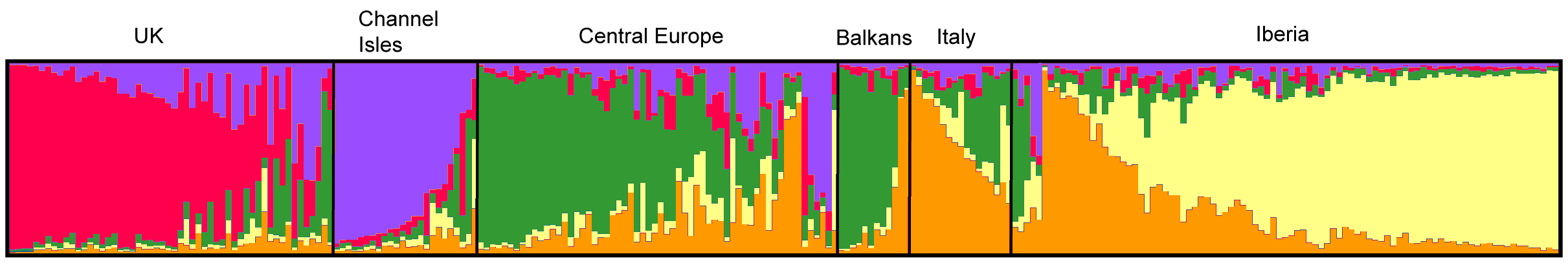


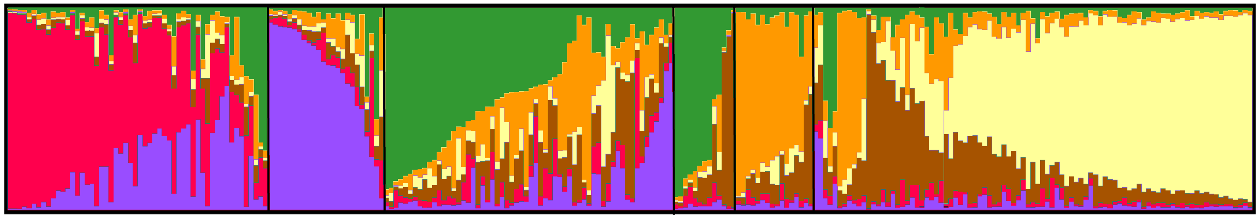


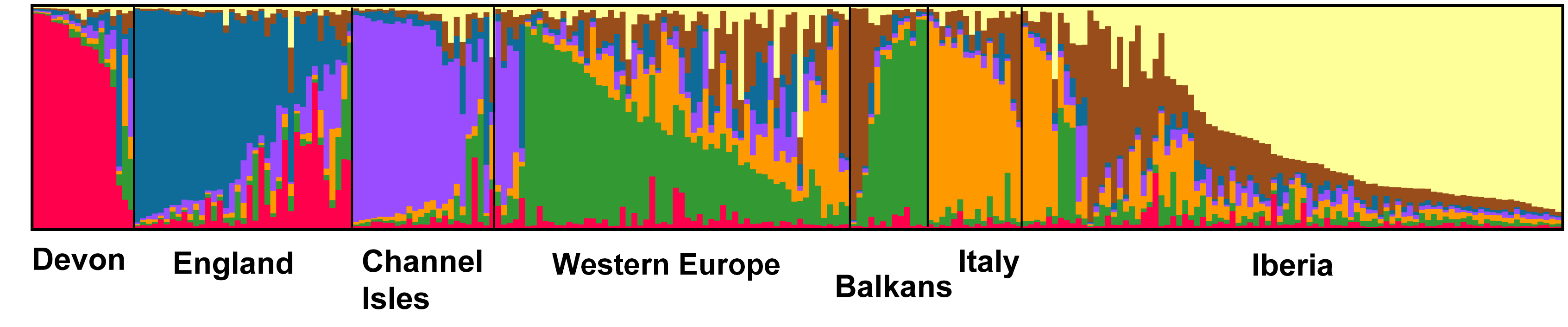


**Figure S2** – **Reclassified binary habitat suitability map based on palaeo-ENMs**. The location of stable areas, where suitable conditions persisted across glacial cycles (between the LIG, LGM and present), is marked in black.


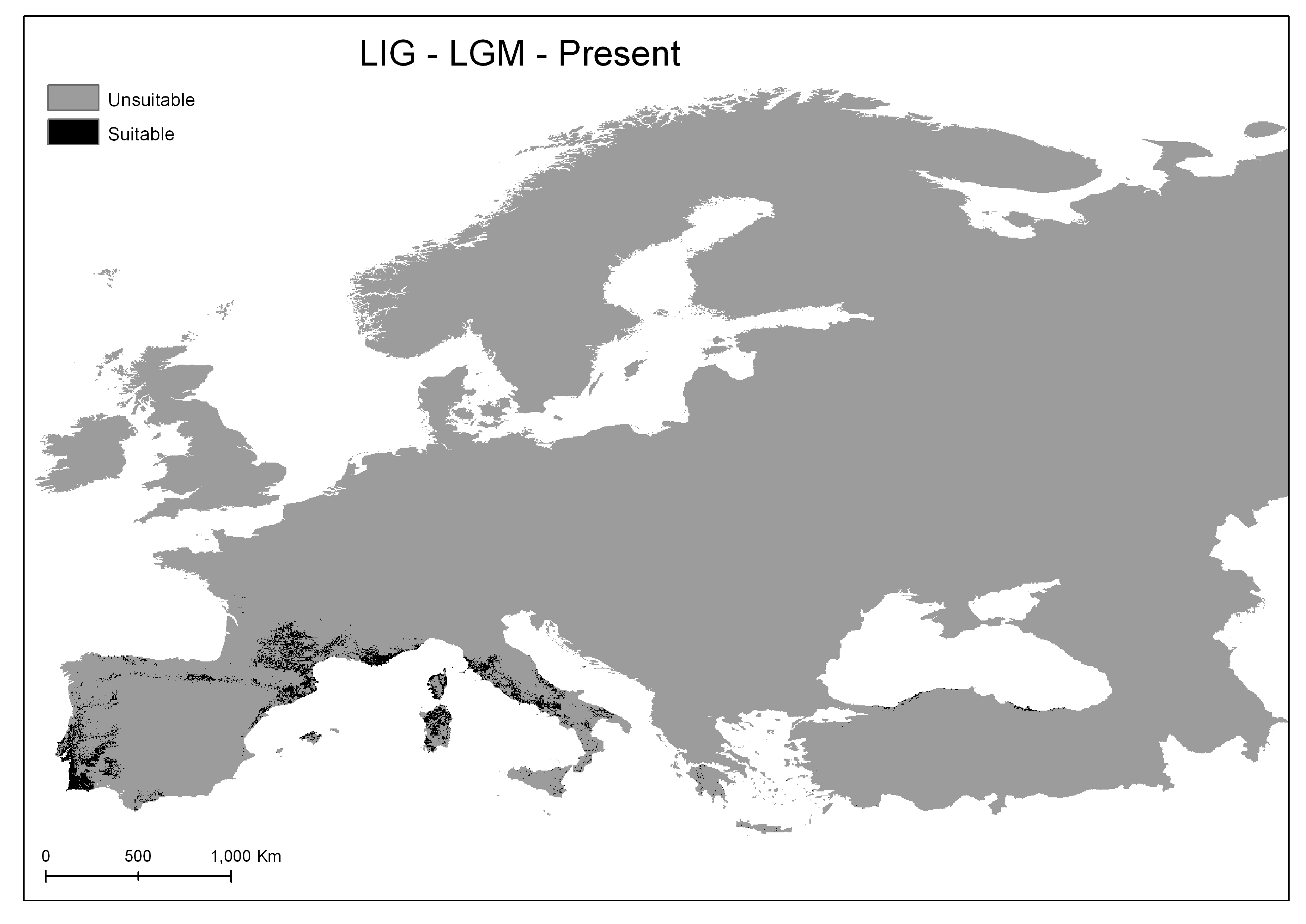


**Figure S3** – **A comparison of the environmental similarity of variables in the LGM to current environmental data.** A) Results of the MESS analysis, with areas that have environmental variables outside the current range marked in pink and red. B) Map showing the most dissimilar environmental variables (those most outside the current range) across Europe. These maps are part of the Maxent modeling output for the CCSM LGM model.


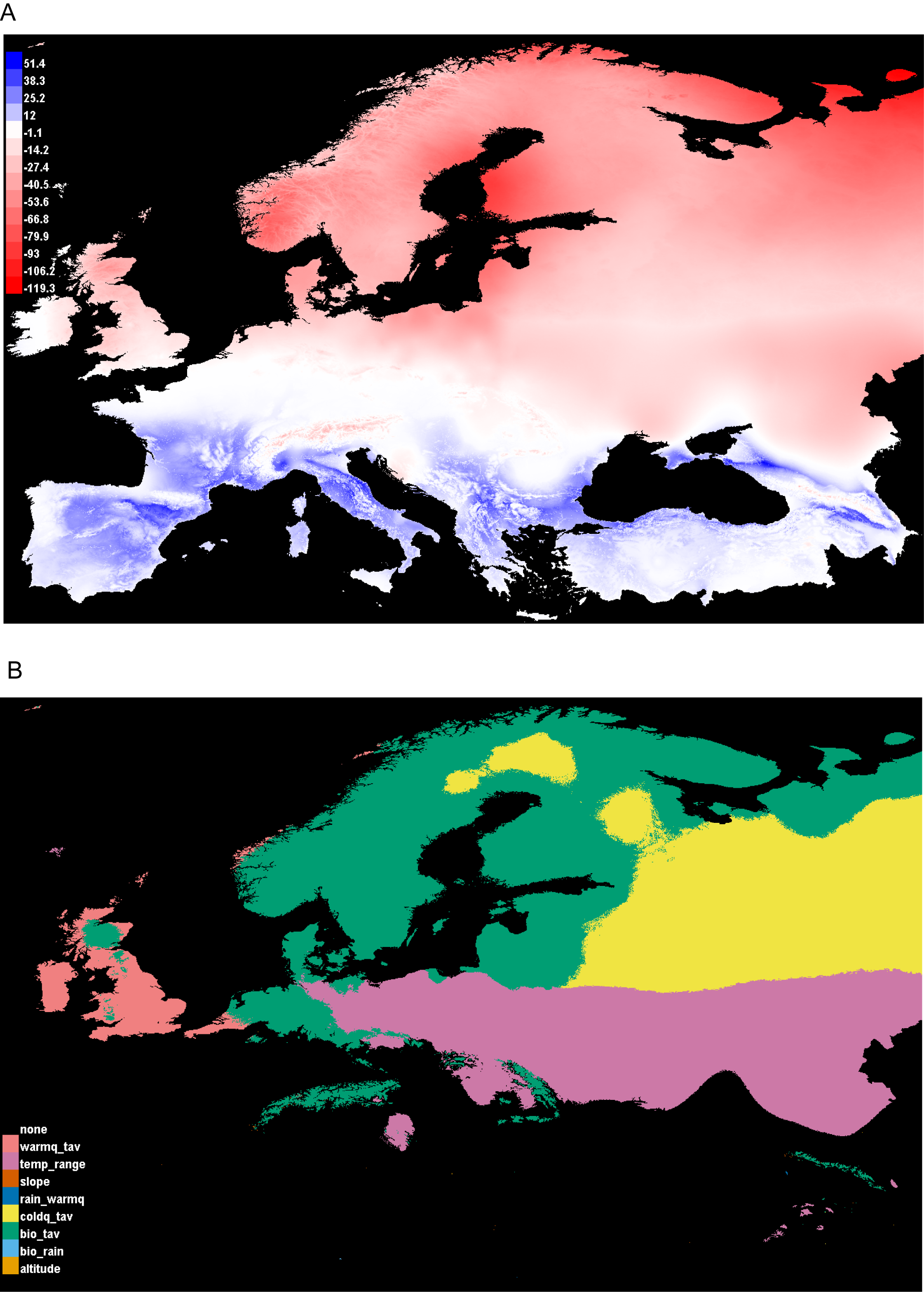


**Figure S4** – **A comparison of the environmental similarity of variables in 2080 to current environmental data.** A) Results of the MESS analysis, with areas that have environmental variables outside the current range marked in pink and red. B) Map showing the most dissimilar environmental variables (those most outside the current range) across Europe. These maps are part of the Maxent modeling output for the CCSM 2080 model.


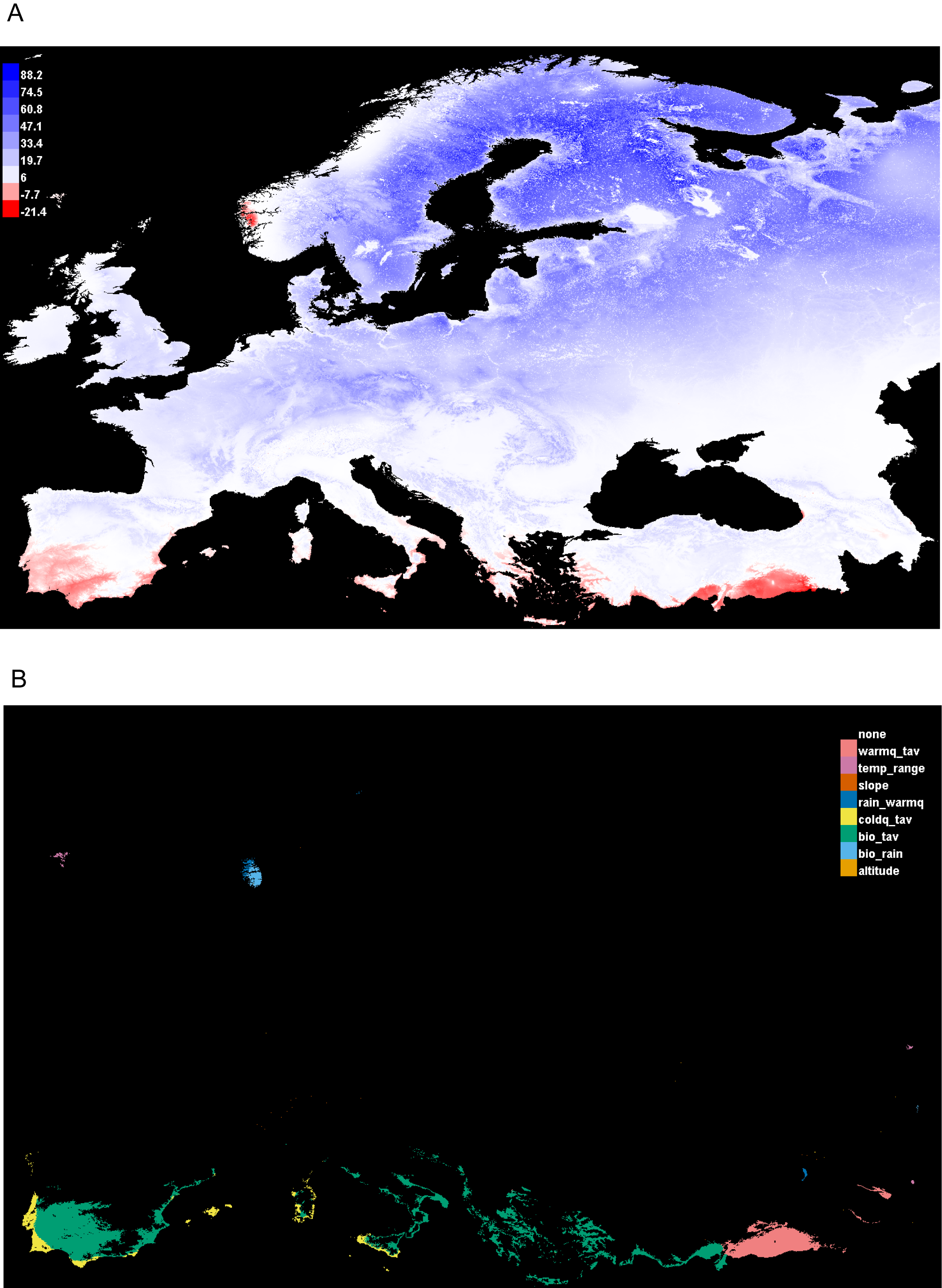


**Figure S5** – **Sampling site-based Species Accumulation Curve** performed at the Cyt *b* haplotype level to determine sampling completeness. Blue dotted line represents the 95% confidence intervals. The resamplig curve reached saturation from around 61 sampling sites.


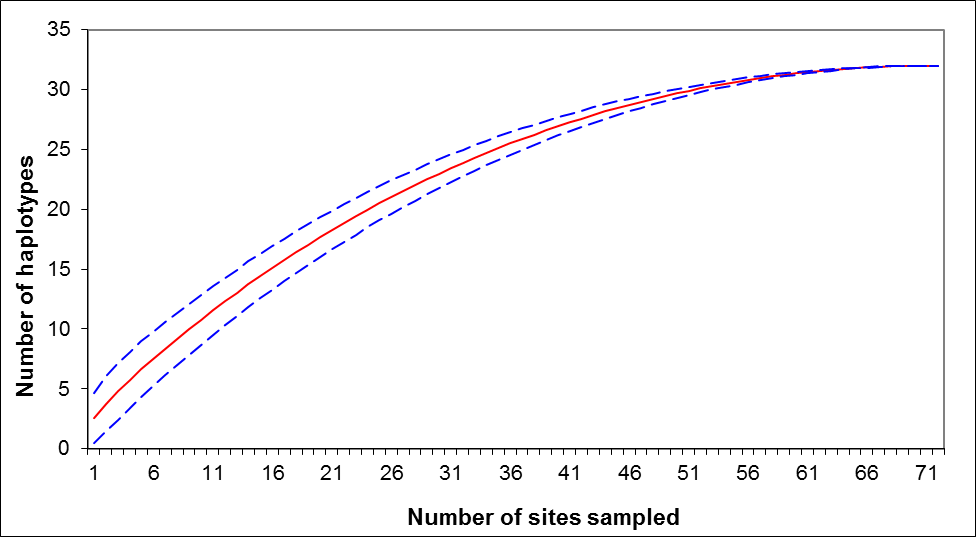


**Figure S6** – **Schematic representation of scenarios compared in the ABC analysis**. A) Scenarios 1.1–1.5 compared in the first analysis (Europe = Western Europe), and the logistic regression of the posterior probabilities of the five scenarios (Y axis) as a function of number of simulated datasets (X axis). Scenarios 1.2, 1.4 and 1.5 all received zero support. Events that occurred after the LGM are marked on the timeline. B) Scenarios 2.1–2.5 compared in the second analysis (grey = unsampled French population), and the logistic regression of the posterior probabilities of the five scenarios. All scenarios received zero support except for 2.1, which received the highest posterior probability.


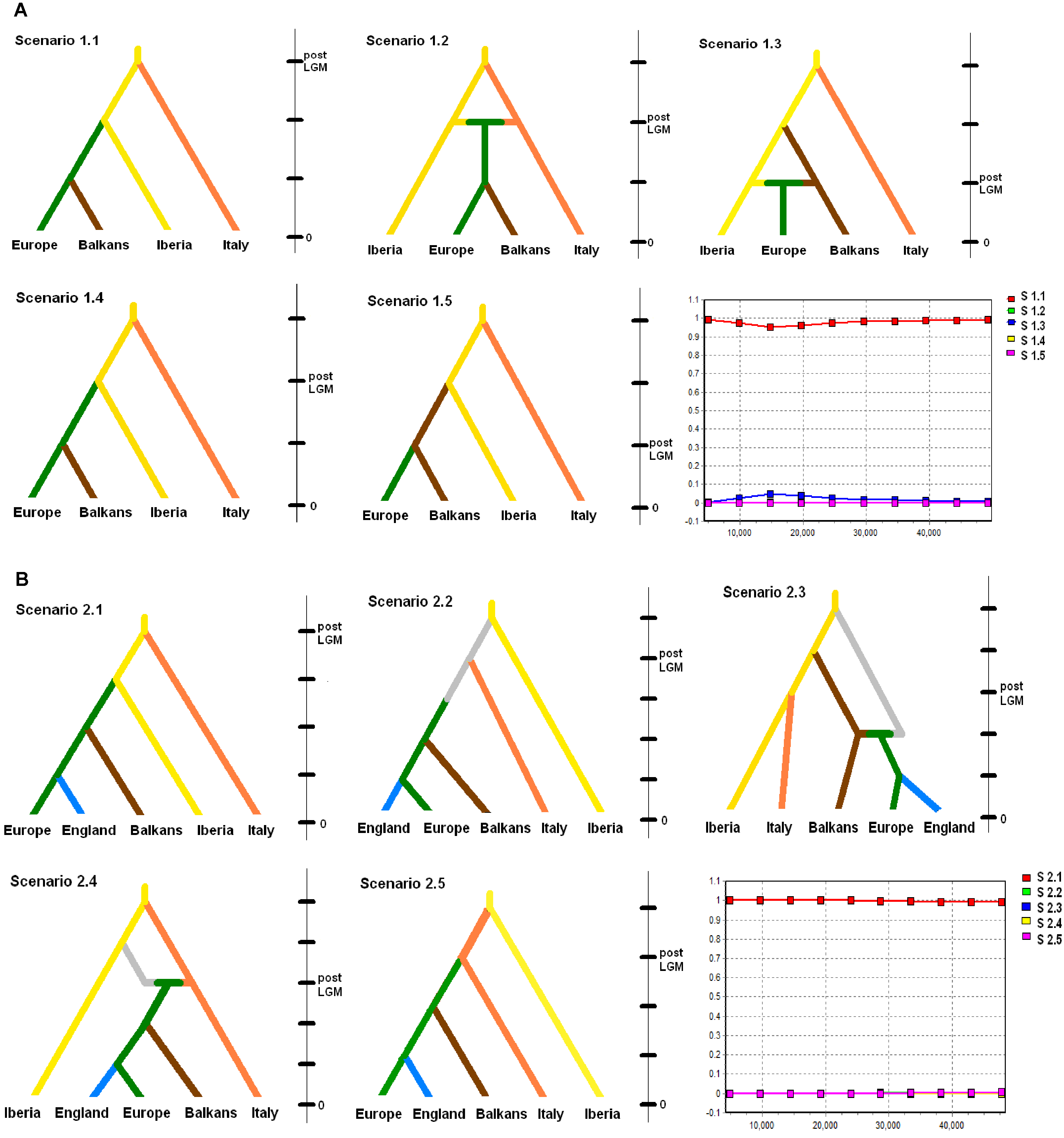


**Figure S7** – **Schematic representation of scenarios compared in the preliminary analysis**. Scenarios P1–P3 included in the preliminary ABC analysis, looking at stepping stone versus long-range or admixture scenarios of post-LGM colonisation of the northern edge of the range (Europe = Western Europe), and the logistic regression of the posterior probabilities of the three scenarios as a function of number of simulated datasets.


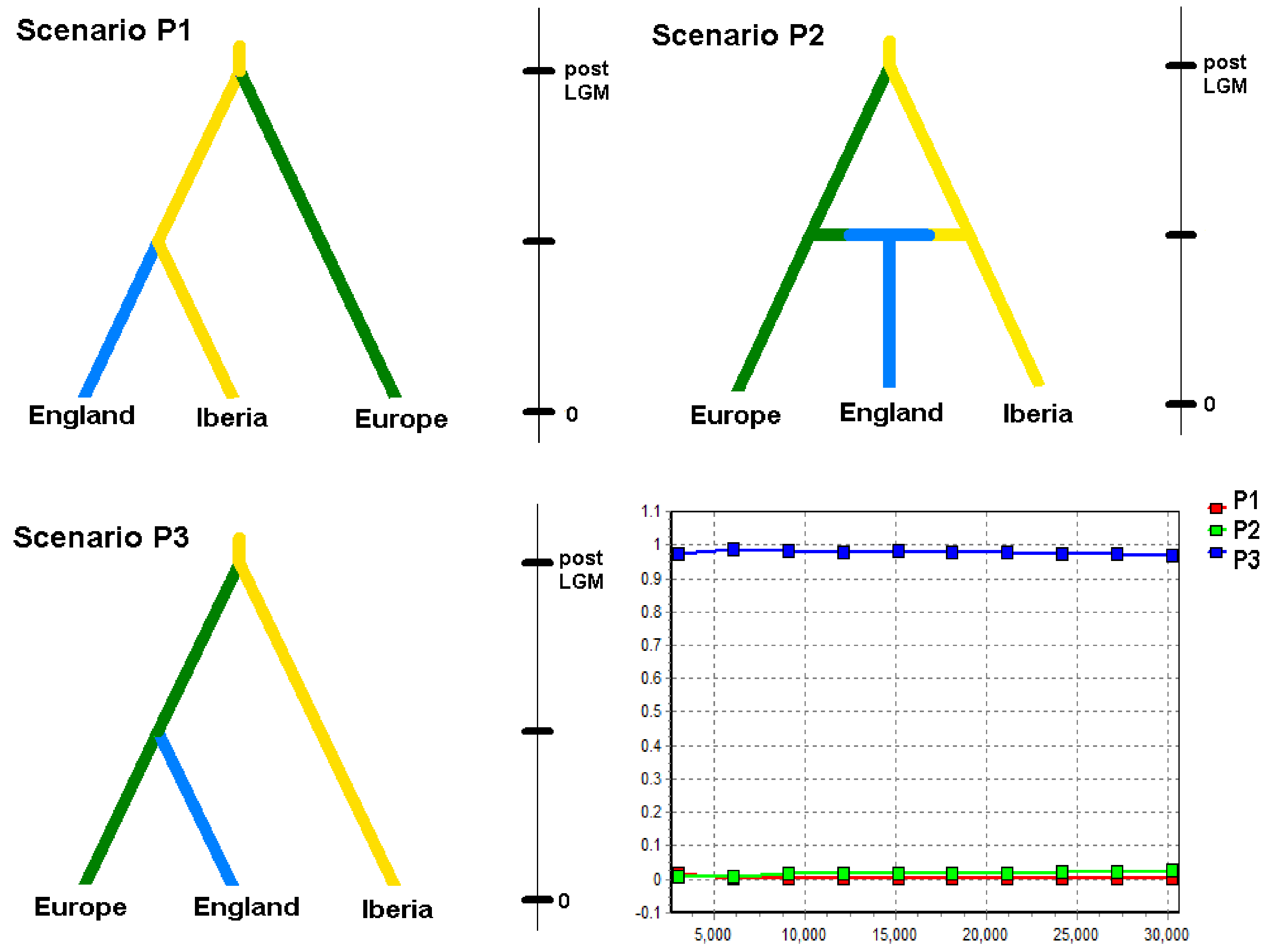


**Figure S8** – **Schematic representation of scenarios compared in the 3^rd^ ABC analysis**. Scenarios 3.1–3.3 looking at changes in the English population size since colonisation (N1– current size of the English population, N2– current size of the Western European population, Bottleneck– bottleneck size of English population during the first few generations following colonisation, and Nc– size of the English population before change in population size at the last few hundreds of generations), and the logistic regression of the posterior probabilities of the three scenarios.


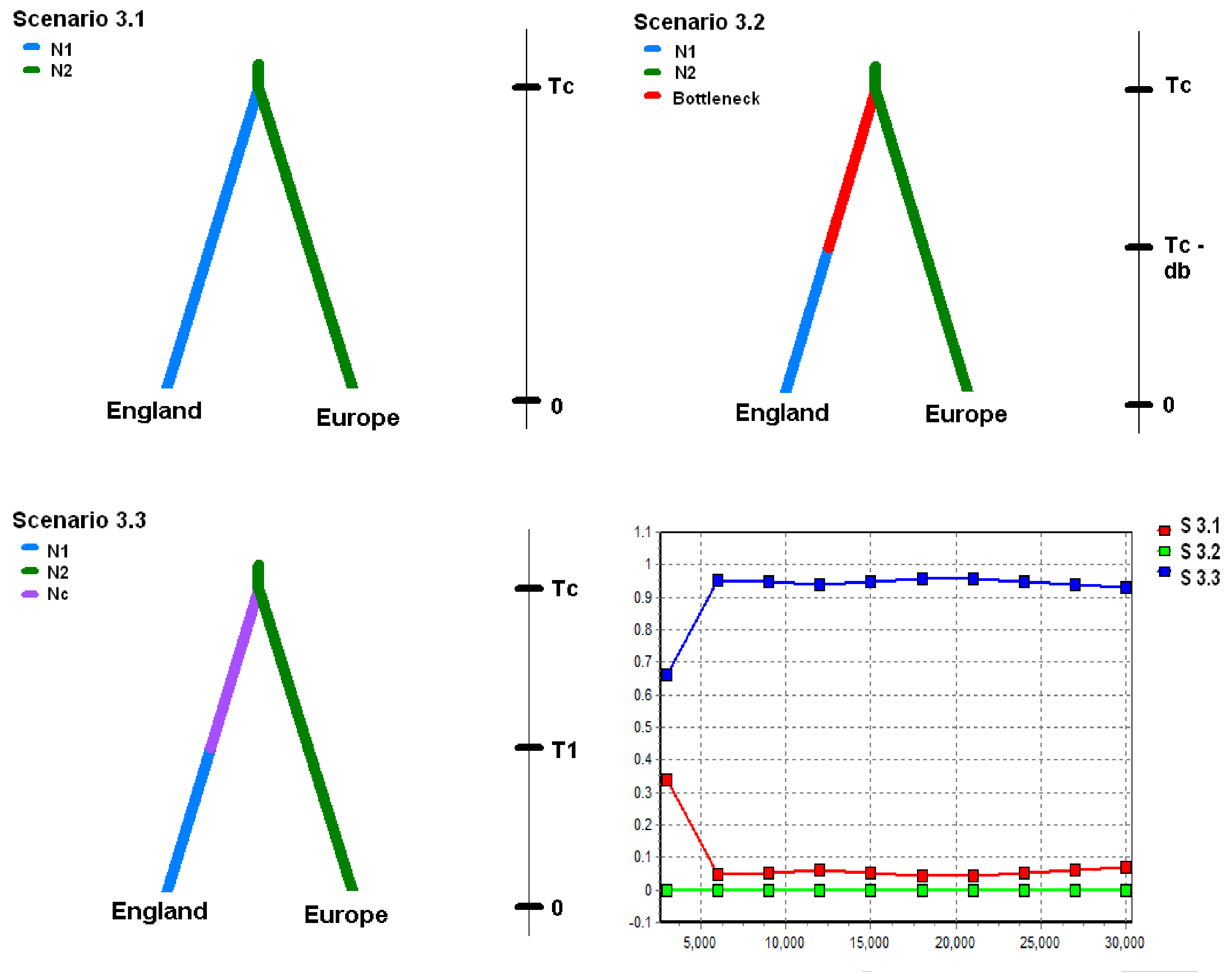

Supplement: Supplementary file 3 [file ele0016-1258-sd3.doc]
